# Dipole Moment‐ and Molecular Orbital‐Engineered Phosphine Oxide‐Free Host Materials for Efficient and Stable Blue Thermally Activated Delayed Fluorescence

**DOI:** 10.1002/advs.202102141

**Published:** 2021-11-21

**Authors:** Soo‐Ghang Ihn, Daun Jeong, Eun Suk Kwon, Sangmo Kim, Yeon Sook Chung, Myungsun Sim, Jun Chwae, Yasushi Koishikawa, Soon Ok Jeon, Jong Soo Kim, Joonghyuk Kim, Sungho Nam, Inkoo Kim, Sangho Park, Dae Sin Kim, Hyeonho Choi, Sunghan Kim

**Affiliations:** ^1^ Samsung Advanced Institute of Technology Samsung Electronics Co., LTD 130 Samsung‐ro, Yeongtong‐gu Suwon‐si Gyeonggi‐do 16678 Korea; ^2^ CSE team Data and Information Technology Center Samsung Electronics Co., LTD 1 Samsungjeonja‐ro Hwaseong‐si Gyeonggi‐do 18448 Korea

**Keywords:** dipole moment, host, organic light‐emitting diode, polarity, thermally activated delayed fluorescence

## Abstract

To utilize thermally activated delayed fluorescence (TADF) technology for future displays, it is necessary to develop host materials which harness the full potential of blue TADF emitters. However, no publication has reported such hosts yet. Although the most popular host for blue TADF, bis[2‐(diphenylphosphino)phenyl]ether oxide (DPEPO) guarantees high‐maximum external quantum efficiency (EQE_max_) TADF devices, they exhibit very short operational lifetimes. In contrast, long‐lifespan blue TADF devices employing stable hosts such as 3′,5‐di(9H‐carbazol‐9‐yl)‐[1,1′‐biphenyl]‐3‐carbonitrile (mCBP‐CN) exhibit much lower EQE_max_ than the DPEPO‐employed devices. Here, an elaborative approach for designing host molecules is suggested to achieve simultaneously stable and efficient blue TADF devices. The approach is based on engineering the molecular geometry, ground‐ and excited‐state dipole moments of host molecules. The engineered hosts significantly enhance delayed fluorescence quantum yields of TADF emitters, as stabilizing the charge‐transfer excited states of the TADF emitters and suppressing exciton quenching, and improve the charge balance. Moreover, they exhibit both photochemical and electrochemical stabilities. The best device employing one of the engineered hosts exhibits 79% increase in EQE_max_ compared to the mCBP‐CN‐employed device, together with 140% and 92‐fold increases in operational lifetime compared to the respective mCBP‐CN‐ and the DPEPO‐based devices.

## Graphical abstract

By using dipole moment‐ and molecular orbital‐engineered host materials which are phosphine oxide‐free, high efficiency and long lifespan can be simultaneously achieved from the organic light‐emitting diodes based on a donor‐acceptor type thermally activated delayed fluorescent emitter.

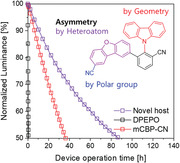

## 1 Introduction

In host–guest organic light‐emitting diodes (OLEDs), the photophysical properties of the light‐emitting molecule are strongly impacted by the host molecule.^[^ ^1^, ^2^, ^3^, ^4^, ^5^ ^]^ Therefore, the host material should be carefully designed or chosen to fulfill the intricate requirements imposed by a given emitter. For instance, to achieve high‐performance OLEDs based on thermally activated delayed fluorescent (TADF) emitters, the polarity of the host material is particularly important owing to the strong charge transfer (CT) character of the excited states in TADF emitters. Host materials with high ground‐state polarity are capable of stabilizing the CT excited states of TADF emitters by the electrostatic interactions between the excited‐state dipole moment of the TADF emitter and the ground‐state dipole moment of the host material.^[^ ^6^, ^7^, ^8^ ^]^ A high‐polarity host can reduce the energy of the lowest singlet excited state (*S*_1_) of the TADF emitter; therefore, the energy gap between *S*_1_ and the lowest triplet excited state (*T*_1_) can be reduced. The emission peaks of the TADF emitters are thus red‐shifted and their reverse intersystem crossing rate (*k*_rISC_) and delayed fluorescence quantum yields (*Φ*_DF_) are enhanced, thus improving the maximum external quantum efficiency (EQE_max_) of the TADF devices.^[^ ^9^, ^10^, ^11^, ^12^ ^]^

Bis[2‐(diphenylphosphino)phenyl]ether oxide (DPEPO) has been the most popular host for blue TADF emitters in recent years,^[^ ^10^, ^11^, ^12^, ^13^, ^14^, ^15^, ^16^ ^]^ demonstrating outstanding EQE_max_ due to its high polarity. However, the band gap of DPEPO is too wide and so its charge (hole in particular) transport abilities is poor and hole injection is intrinsically hindered, resulting in a high driving voltage, poor electrochemical stability, and relatively short device operation lifetime. Moreover, DPEPO consists of phosphine oxide moieties which induce faster degradation.^[^ ^17^, ^18^, ^19^ ^]^ Hence, no studies have reported long operational lifetimes of DPEPO‐based TADF devices. On the other hand, at the expense of efficiency, several highly stable blue TADF devices have been obtained with stable hosts such as 3,3′‐di(9H‐carbazol‐9‐yl)‐1,1′‐biphenyl (mCBP) and 3′,5‐di(9H‐carbazol‐9‐yl)‐[1,1′‐biphenyl]‐3‐carbonitrile (mCBP‐CN).^[^ ^19^, ^20^, ^21^ ^]^

Herein, we introduce alternative host materials that are capable of stabilizing the CT excited states of TADF emitters by their relatively high ground‐state polarity, while simultaneously providing long OLED operational lifetimes owing to their narrower band gaps, better charge transporting ability, and greater material stability compared to phosphine oxide‐based DPEPO. The novel host material‐based blue TADF devices exhibit similar efficiency to the DPEPO‐based devices, and are as stable as those using mCBP‐CN. The simultaneous achievement of high efficiency and stability of these high‐polarity and phosphine oxide‐free compounds makes them suitable alternative host materials for DPEPO‐ and mCBP‐CN.

## 2 Results

### 2.1 Molecular Asymmetry and Polar Groups Introduce High Polarity to Phosphine Oxide‐Free Host Materials

**Figure 1:**
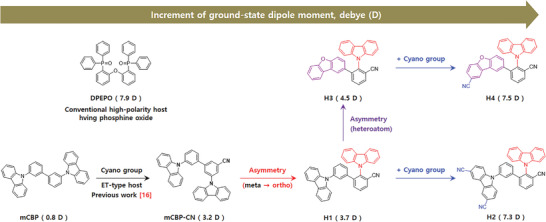
Design strategies for high‐polarity host materials and their chemical structures. Strategies for increasing the polarity of host materials by means of increasing structural asymmetry and their examples (H1–H4, derivatives of mCBP‐CN). The values in the brackets are the calculated ground‐state dipole moments, *μ*_GS_, in debye (D).

The ability to stabilize the CT excited state of TADF emitters primarily depends on the geometry and electrostatic properties of the host materials. In particular, the dipole moment serves as a convenient scale for polarity because of its dominant contribution to the electrostatic interactions between the emitter and host molecules. With the aim of designing a phosphine oxide‐free host with high polarity, we introduced asymmetry and polar groups into the molecular backbone of a leading molecule to carefully control the dipole moment. As a leading molecule, we considered mCBP‐CN (**Figure** **1**), which is an electron‐transporting host derived from the well‐known mCBP by attaching a polar cyano (CN) group to one of the two phenyls. The asymmetrically attached CN group increased the ground‐state dipole moment, *μ*_GS_, to 3.4 D (debye, where 1 D ≈ 3.34 × 10^−30^ C⋅m), although its *μ*_GS_ was still below that of DPEPO.

**Figure 2:**
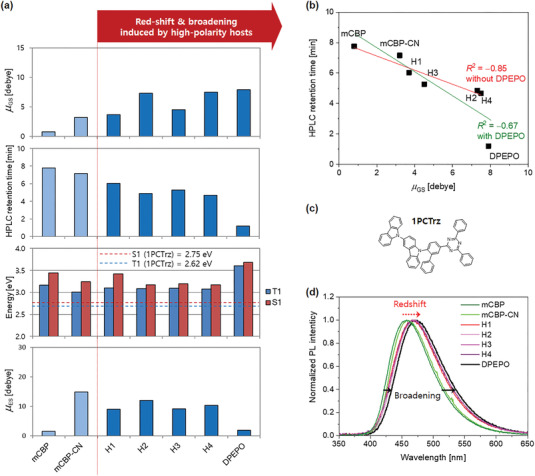
Increased host polarity correlates with reduced HPLC retention time and redshifted and broadened photoluminescence (PL) spectra. a) Calculated values of ground‐state dipole moments, *μ*_GS_ (first panel); HPLC retention time (second panel); *T*_1_ and *S*_1_ energies (third panel) and calculated values of excited‐state dipole moments; *μ*_ES_ (fourth panel) of the hosts. b) Correlation between ground‐state dipole moment and HPLC retention time. c) Chemical structure of TADF emitter, 1PCTrz. d) Photoluminescence spectra of 1PCTrz in the various host materials. The compositional ratio of host/1PCTrz is 85/15 by volume and the film thickness is 50 nm for all the films. Compared to the emission peaks of 1PCTrz in mCBP and mCBP‐CN, those in H1–H4 and DPEPO are redshifted (red dotted arrow) and broadened (black solid arrows). *μ*_GS_, *μ*_ES_, *T*_1_, and *S*_1_ are represented as the values corresponding to the minimum energy structure for each host (see also Figure S9, Supporting Information).

By modifying the asymmetry and polar groups of mCBP‐CN, we obtained four novel host materials, H1–H4, with improved *μ*_GS_ values (**Figure** **2**a, first panel). H1 was obtained by adjusting the bonding position of one of the carbazole groups of mCBP‐CN from meta to ortho to induce asymmetry, thereby increasing *μ*_GS_ to 3.7 D. H2 was obtained by attaching two additional CN groups to the second carbazole group of H1, leading to a much higher *μ*_GS_ of 7.3 D. H3 was achieved by substituting the second carbazole group of H1 with a benzofuran group; the introduction of a heteroatom (oxygen) slightly improved the *μ*_GS_ value (4.5 D) over that of H1. Finally, an additional CN‐functionalization on the dibenzofuran group of H2 resulted in H4, which exhibited a higher *μ*_GS_ of 7.5 D. Notably, the *μ*_GS_ values of H2 and H4 are close to the value of 7.9 D for DPEPO.

To experimentally compare the ground‐state polarities of the host materials, we measured their retention times with high‐performance liquid chromatography(HPLC). The elution order of solutes in HPLC is governed by polarity,^[^ ^22^ ^]^ therefore, HPLC measurements provide details about the polarity of materials. H1–H4 and DPEPO showed shorter HPLC‐retention times than mCBP and mCBP‐CN (Figure 2a, second panel), indicating that H1–H4 and DPEPO have higher ground‐state polarities. DPEPO had the shortest HPLC‐retention time. Consequently, the HPLC‐retention times can scale the examined host materials as well as the ground‐state dipole moments do but they have an inverse tendency. Figure 2b shows such correlation and we obtained the correlation coefficient *R* ^2^ of −0.67 when considering DPEPO and −0.85 without DPEPO.

To explore their suitability as host materials for TADF emitter molecules, we investigated whether the excited‐state energies (*S*_1_ and *T*_1_) of H1–H4 would be high enough to confine excitons within a given blue TADF emitter, 1PCTrz which has a typical structure of twisted intramolecular CT (TICT) molecules (Figure 2c).^[^ ^23^ ^]^ Our calculations indicated that H1–H4 had sufficiently higher *S*_1_ and *T*_1_ values (Figure 2a, third panel) than the given TADF emitter. To verify the ground‐state polarities of the host materials and investigate their effect on the photophysical properties of the TADF emitter, we compared photoluminescence (PL) spectra of thin films composed of the given TADF emitter and host material.

According to the work of Lippert et al.,^[^ ^24^ ^]^ polar solvents can redshift the emission of TICT molecules which are often employed as TADF emitters. In addition, the polarity of the solvents changes the emission state of TICT molecules between a locally excited state and CT state by changing the twist angle between the electron accepting moiety and electron donating one, because they are not used again in the paper except their combination, donor‐acceptor (D‐A).^[^ ^25^ ^]^ When fabricated with H1–H4 and DPEPO, 1PCTrz also exhibited redshifted (8–14 nm) and broadened (7–9 nm) PL emission compared to that with mCBP and mCBP‐CN, as shown in Figure 2d. The PL peak wavelengths and full‐width at half maximum (FWHM) values for all the tested host:1PCTrz films are summarized in **Table** **1**.

**Table 1:** Calculated dipole moments of various hosts and photophysical properties of 1PCTrz‐doped host films

Host	*μ*_GS_ ^a)^ [D]	*μ*_ES_ ^b)^ [D]	*μ*_ES_^exp^ ^c)^ [D]	*λ*_PL_ ^d)^ [nm]	FWHM^e)^ [nm]	Quantum Yield [%]			*τ*_PF_ ^f)^ [ns]	*τ*_DF_ ^g)^ [µs]	*k*_rISC_ ^h)^ [×10^5^ s^−1^]
						Delayed	Prompt	Total			
mCBP	0.8	1.5	4.3	457	80	23.8	39.9	63.7	11.3	18.8	0.85
mCBP‐CN	3.2	14.9	14.8	458	79	10.9	52.0	62.9	10.9	10.3	1.12
H1	3.7	9.1	14.4	467	87	54.0	18.8	72.8	12.6	27.2	1.43
H2	7.3	12.0	19.0	470	87	37.9	37.0	74.9	11.9	15.0	1.35
H3	4.5	9.2	14.2	469	88	54.4	25.7	80.1	12.7	24.2	1.29
H4	7.5	10.3	17.1	466	88	47.3	32.0	79.3	11.7	20.7	1.19
DPEPO	7.9	1.9	–	471	88	46.5	32.5	79.0	14.0	13.3	1.82

^a)^ *μ*_GS_;

^b)^ *μ*_ES_: Ground‐state dipole moment and excited‐state dipole moment obtained from the density functional theory (DFT) calculations at the B3LYP/def2‐SVP level through the use of the TURBOMOLE 7.5 program suite. The excited‐state dipole moments were calculated at the spin‐component‐scaled (SCS)‐CC2 level of the second‐order approximate coupled‐cluster singles and doubles (CC2) calculations with the first‐excited singlet state geometries which are optimized by the TD‐B3LYP level without any symmetry constraints. The resolution‐of‐the‐identity approximation was used in all calculations. See Experimental Section for details;

^c)^ *μ*_ES_^exp^: The excited‐state dipole moment obtained by using the Lippert–Mataga method;

^d)^ *λ*_PL_: Peak wavelength of the PL peak;

^e)^ FWHM: Full‐width at half maximum;

^f)^ *τ*_PF_: Prompt fluorescence lifetime;

^g)^ *τ*_DF_: Delayed fluorescence lifetime;

^h)^ *k*_rISC_: Rate constant of reverse intersystem crossing.

### 2.2 High Polarity Hosts Enhance the Delayed Fluorescence Quantum Yield

The primary purpose of employing high‐polarity hosts for TADF devices is to deduce their potential for stabilizing the CT excited states of TADF emitters, in the same manner as DPEPO. The improvement of the EQE_max_ of DPEPO‐based TADF devices originates from the enhanced reverse intersystem crossing rate, *k*_rISC_, and delayed fluorescence quantum yield, *Φ*_DF_, of the TADF molecules owing to the high‐polarity DPEPO matrix. Based on the results of PL experiments in Figure 2d, we expected H1–H4 to have the same effect as DPEPO does. We obtained *Φ*_DF_ and *k*_rISC_ values for our new hosts, DPEPO, mCBP, and mCBP‐CN from PL quantum yield (PLQY) and transient PL experiments as shown in Table 1. It is found that *Φ*_DF_ of 1PCTrz:DPEPO is lower than that of 1PCTrz:H1, H3, and H4 films in spite of its largest *k*_rISC_. Here, *k*_rISC_ were calculated using the relations, *k*_rISC_ = *Φ*_DF_/(*τ*_PF_ *τ*_DF_ *k*_ISC_ *Φ*_PF_) and *k*_ISC_ = *Φ*_PF_/*τ*_PF_[1/*Φ*_PF_ − 1/(*Φ*_DF_ + *Φ*_PF_)] according to Zeng et al.^[^ ^26^ ^]^ 1/*τ*_DF_ and *k*_rISC_ are not in a simply linear relationship due to their dependence on other quantities. We thus observe that *τ*_DF_ in the DPEPO matrix is shorter than the cases using H1–H4, which leads to a relatively lower *Φ*_DF_ compared to H1, H3, and H4.

Comparing the effect of the hosts, H1–H4, with that of mCBP and mCBP‐CN, 1PCTrz exhibited higher *Φ*_DF_ and *k*_rISC_ values when hosted in H1–H4 than in mCBP and mCBP‐CN as shown in Table 1. This result is ascribed to the high polarity of H1–H4, as indicated by the enhanced *μ*_GS_ values. However, *Φ*_DF_ did not monotonically increase with *μ*_GS_, even when comparing hosts with the same core structure. H2 and H4 which have *μ*_GS_ values near 7 D exhibited lower *Φ*_DF_ values than H1 and H3. It should be noted that the dipole moment of the host has two sides in relation with *Φ*_DF_. In the ground state, a high dipole moment enhances delayed fluorescence; while in the excited state, it facilitates exciton quenching through intermolecular CT owing to interactions with the emitter dipoles in the CT excited state.^[^ ^8^ ^]^ The excited‐state dipole moments obtained from theoretical calculations (*μ*_ES_) and experiments based on the Lippert–Mataga equation^[^ ^8^, ^27^, ^28^, ^29^ ^]^ (*μ*_ES_^exp^) are given in Figure 2a and Table 1. *μ*_ES_ in comparison with *μ*_GS_ can be resolved in terms of natural transition orbitals (NTOs) for the CT excited states. We display sets of NTOs for the host molecules as well as the direction of *μ*_GS_ in Figure S1, Supporting Information. *μ*_ES_ is generally determined by the relative orientation of *μ*_GS_ and the additional dipole moment produced in the excited state. H2 and H4, which have high *μ*_GS_ values, exhibit higher *μ*_ES_ values compared with H1 and H3, because the sum of *μ*_GS_ and the additional CT‐dipole tends to increase in its magnitude. As a result, H2 and H4 exhibit relatively low *Φ*_DF_ values compared with H1 and H3. This implies that their CT‐character is strengthened in the excited state, and enhances exciton quenching. The formation of intermolecular CT complexes depends on the energy‐level structures of the host and emitter molecules. Although *μ*_ES_ value of DPEPO is below those of H1–H4, its *Φ*_DF_ value is not the highest one. Because of extremely wide bandgap of DPEPO, it is not probable to form intermolecular CT complexes in the excited state of DPEPO.

The effect of these excited‐state dipole moments on the performance of TADF devices is discussed later. Considering the higher *Φ*_DF_ and *k*_rISC_ values and redshifted and broadened emission of 1PCTrz in the novel host materials with high‐dipole moments (H1–H4), we expect them to be strong candidates for replacing both high‐polarity hosts such as DPEPO and stable hosts such as mCBP‐CN.

### 2.3 Ground‐State Characteristics of the High‐Dipole Moment Host Materials

**Figure 3:**
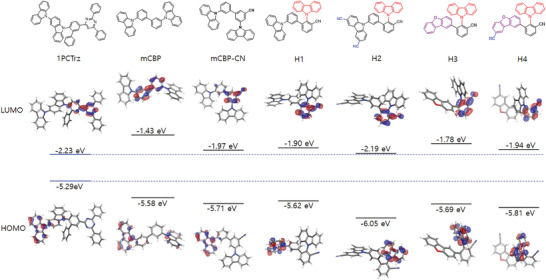
Ground‐state characteristics of 1PCTrz and various hosts. Displayed are contours of HOMOs and LUMOs and their energy levels. The HOMO energy levels of the hosts are all much lower than that of 1PCTrz, while the LUMO energy levels of the hosts are slightly shallower than that of 1PCTrz, except for that of mCBP, which is considerably higher.

Although donor–acceptor (D–A) type TADF emitters have bipolar characteristics, they largely show hole‐transporting behavior when used with electron‐transporting host materials such as mCBP‐CN.^[^ ^19^ ^]^ TADF devices typically have high emitter/host compositional ratios compared to other technologies such as a fluorescent and phosphorescent OLEDs; therefore, to provide high efficiency and stability, an electron‐transporting host such as mCBP‐CN is required, which forms a mixed‐host‐like emitting layers with hole‐transporting TADF emitters. In this work, the employed TADF emitter, 1PCTrz, also exhibits hole‐transporting behavior rather than electron‐transporting behavior, although it is basically a bipolar D–A molecule as shown in **Figure** **3**. Thus, the host should exhibit electron‐transporting properties.

The designed hosts, H1–H4, all exhibit electron‐transporting behavior (Figure 3). According to our calculations, H1–H4 have the comparable lowest unoccupied molecular orbitals (LUMOs) to that of 1PCTrz, yet much lower highest occupied molecular orbitals (HOMOs) than both 1IPCTrz and mCBP, in a similar manner to mCBP‐CN. In Figure 3, the locations of frontier orbitals are unaffected by changing the position of the carbazole from meta to ortho in the design of H1 based on mCBP‐CN. However, the orbital energies of H1 increases slightly compared to mCBP‐CN, because the bond connecting phenyl and CN‐substituted phenyl should rotate toward a different configuration due to steric hindrance as depicted in Figure S2, Supporting Information, resulting in slight increases of orbital energies of H1 compared to mCBP‐CN. In H1, the CN‐substituted phenyl‐bridges correspond to more electron accepting moiety and hence not only accommodating the locus of LUMO of H1 (Figure 3) but also resulting in deeper occupied MO levels for attached carbazole moiety. The HOMO is thus observed on the carbazole attached to the unsubstituted phenyl‐bridge in H1. By the addition of the withdrawing group, ‐CN, (H1 (CN‐added) in Figure S3, Supporting Information), the HOMO becomes even further lowered and hence appear on the carbazole attached to the CN‐substituted phenyl‐bridge. This relocation of the locus of HOMO accompanies a drastic decrease in the HOMO energy of 0.31 eV. Further addition of a CN‐group (H1 (2CN‐added) in Figure S3, Supporting Information) lowers the HOMO only by 0.1 eV. Note that in H3, the HOMO locus is already on the carbazole and an extra CN‐group in H4 has lowered the HOMO by about 0.1 eV in Figure 3, which is quite similar in going from H1 (CN‐added) to H1 (2CN‐added). Our previous work^[^ ^19^ ^]^ revealed that electron‐transporting hosts can form mixed‐host‐like emitting layers with highly doped TADF emitters, which can improve the stability of TADF devices. Note that H1 and H2, which share the same core structure show different HOMO and LUMO energy levels. Specifically, H2 has lower‐lying HOMO and LUMO energy levels than H1, which indicates that H2 has stronger electron‐transporting behavior. H4 also has lower‐lying HOMO and LUMO energy levels than H3 and allows stronger electron‐transport than H3. We measured the HOMO energy levels of H1–H4 with a photoelectron spectrophotometer, AC‐3 (RIKEN KEIKI Co., LTD.), and the variation of experimental HOMO energy levels is consistent with that of calculated ones, as demonstrated in Figure S4, Supporting Information. It is interesting to compare H2 with H1 and H4 with H3, particularly from the view point of stability of the devices, which we will discuss in the following section.

### 2.4 Device Characteristics

**Figure 4:**
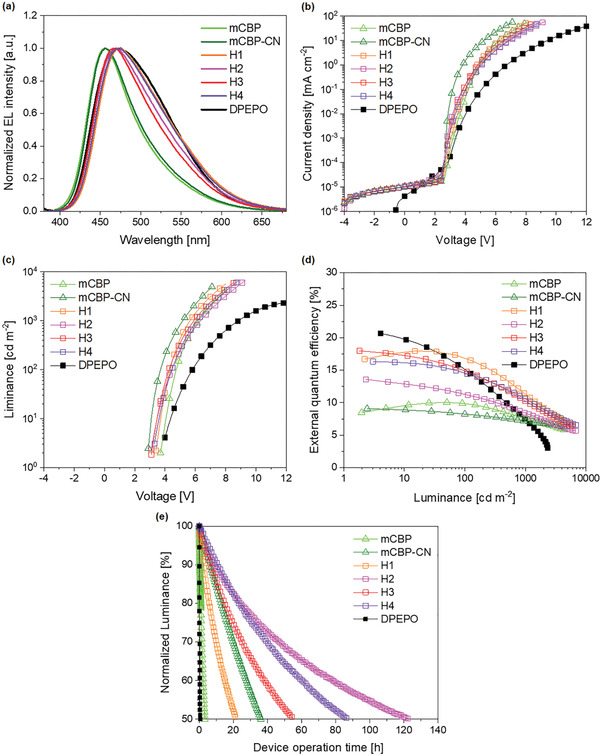
Characteristics of OLEDs. a) Electroluminescence spectra of the OLEDs at 500 cd m^−2^. b) Current density–voltage characteristics, c) luminance–voltage characteristics, and d) EQE of the OLEDs as a function of luminance. e) Normalized luminance of the OLEDs as a function of operation time at a constant current density. The initial luminance was 500 cd m^−2^. The compositional ratio of host/1PCTrz is 80/20 by volume and the thickness is 40 nm for all the emitting layers.

The device characteristics of the tested OLEDs are shown in **Figure** **4** and their performances are summarized in **Table** **2**. The OLEDs were fabricated with the following layer stacks: indium tin oxide (ITO)/p‐doped (3 wt%, NDP series, see the Device Fabrication and Measurement section) N‐([1,10‐biphenyl]‐4‐yl)‐9,9‐dimethyl‐N‐(4‐(9‐phenyl‐9H‐carbazol‐3‐yl) phenyl)‐9H‐fluoren‐2‐amine (BCFA) (10 nm)/BCFA (135 nm)/ 2,2′‐di(9H‐carbazol‐9‐yl)‐1,1′‐biphenyl (oCBP) (10 nm)/host:1PCTrz (40 nm)/2,8‐bis(diphenylphosphine oxide) dibenzofuran (DBFPO) (10 nm)/co‐deposited NET:NDN series (5:5 by volume, 30 nm, see the Device Fabrication and Measurement section)/Al. The device structure is the same as the OLED device reported by S. Hong et al.,^[^ ^30^ ^]^ except for the emitting layer and electron transport layer.

**Table 2:** Device performance of 1PCTrz‐based OLEDs employing various hosts

Host	Voltage^a)^ [V]	Current efficiency^a)^ [cd A^−1^]	Power efficiency^a)^ [lm W^−1^]	EQE^b)^ [%]			LT50^c)^ [h]
				Max	500 cd m^−2^	1000 cd m^−2^	
mCBP	5.60	12.85	7.22	10.0	8.4	7.5	3.3
mCBP‐CN	4.58	11.86	8.14	9.1	7.5	7.2	36.2
H1	5.09	26.68	16.49	17.9	13.4	11.3	21.7
H2	5.60	20.52	11.51	13.6	9.5	8.4	122.4
H3	5.25	24.73	14.80	18.0	11.9	10.3	55.1
H4	5.47	26.32	15.13	16.3	12.1	10.6	86.9
DPEPO	7.62	20.41	8.42	21.1	9.9	7.5	0.6

^a)^ At 500 cd m^−2^;

^b)^ EQE: External quantum efficiency;

^c)^ Time until the luminance decreased to 50% of the initial luminance of 500 cd m^−2^.

The normalized electroluminescence (EL) spectra (Figure 4a) of the TADF devices look similar to the normalized PL spectra (Figure 2d), with only small differences ascribed to the variation of the exciton profiles in the emitting layers and weak micro‐cavity effects in multilayered OLEDs.^[^ ^31^, ^32^, ^33^ ^]^ Thus, in the same manner as the PL spectra, the EL spectra of the TADF devices employing high‐polarity hosts (H1–H4, DPEPO) were slightly redshifted and broader than those of the OLEDs based on mCBP or mCBP‐CN. This result indicates that host polarity has the same effect in the electrically driven multilayered OLEDs as in the optically pumped single‐layer thin films.

Figure 4b,c shows the current density–voltage and luminance–voltage characteristics of the OLEDs, respectively. The DPEPO‐based OLED, which has the widest bandgap (5.43 eV, calculated) showed the highest device resistance; while mCBP‐CN has the second narrowest bandgap (3.74 eV, Figure 3) shows the smallest device resistance. Although H1 has the narrowest bandgap (3.72 eV, Figure 3), the device resistance of H1 is higher than that of mCBP‐CN, because the difference in device resistance is very small, yet mCBP‐CN has a lower LUMO, which may facilitate charge transport within the highly doped hole‐transporting TADF emitter.^[^ ^19^ ^]^

Figure 4d shows the EQE–luminance characteristics of the OLEDs. The devices employing H1–H4 exhibit higher EQE_max_ of 13.6–18.0% than the mCBP‐based (10.0%) and mCBP‐CN‐based (9.1%) OLEDs. These high EQE_max_ values can be attributed to the increased delayed fluorescence of 1PCTrz in H1–H4, as these hosts provide highly a polar environment. The H3:1PCTrz film exhibits the highest *Φ*_DF_ among the 1PCTrz‐doped films employing phosphine oxide‐free hosts (H1–H4, mCBP, and mCBP‐CN) and consequently the H3:1PCTrz‐based OLED exhibits the highest EQE_max_ (18.0%). Notably, the EQE_max_ of the H3:1PCTrz‐based OLED is the closest to that of the DPEPO:1PCTrz‐based OLED (21.1%). Furthermore, at practical brightnesses (500 or 1000cd m^−2^, for example), the EQE of the H3:1PCTrz‐based OLED exceeds that of the DPEPO:1PCTrz‐based OLED as efficiency roll‐off is suppressed in the former. Despite the short delayed fluorescence lifetime (*τ*_DF_) of the DPEPO system, DPEPO:1PCTrz‐based OLEDs exhibit significant efficiency roll‐off due to severe charge imbalance caused by DPEPO.^[^ ^19^ ^]^ Although *τ*_DF_ for the H1–H4 systems were longer than those for the DPEPO system, the devices based on H1–H4 showed higher EQEs at a high luminance (1000 cd m^−2^). This is attributed to the improved charge balance in the emitting layer, where a lower LUMO level of H1–H4 leads to increased injection of electrons. The initial balance and the resulting exciton distribution can be shifted as a result of degradation during the device operations. We can recognize it through the observation of EL shifts. All the EL devices in this study showed redshift after the device operation for the device stability test. It indicates that the recombination sites of the devices are all shifted toward the electron blocking layer after the device operation, which means that the loss of hole transporting ability is one of the main device degradation factors. The increment of the color coordinate (CIEy) indicating the redshift is presented in the Figure S5, Supporting Information.

It is noteworthy that the EQE_max_ values for the devices based on H1–H4 varied consistently with *Φ*_DF_, indicating that the effects of host polarity observed in the optical experiments are also applicable to the electrical devices. Among H1–H4, H3 exhibits the highest EQE_max_, despite its relatively low *μ*_GS_ (4.5 D, Table 1). This can be explained by low *μ*_ES_^exp^ of H3 compared with that of H2 and H4 (14.2 D vs 19.0 D and 17.1 D). We believe that excitons from the 1PCTrz can be significantly quenched by hosts (H2 and H4) as their excited‐state polarities are higher than that of H3, inducing easier formation of intermolecular CT complexes. In contrast, in H3, 1PCTrz can avoid serious exciton quenching owing to relatively low *μ*_ES_^exp^ of H3 which affects the influence of the excited‐state dipole field of the host.^[^ ^8^ ^]^ The DPEPO‐based OLED still has the greatest EQE_max_ (21.1%), because the extremely wide band gap of DPEPO makes it difficult for DPEPO to reach the excited state or form an intermolecular CT complexes with other molecules. For efficiency, H1 and H3 are probably the most promising candidates as alternative hosts for blue TADF devices.

To investigate the influence of the host materials on the device stability, the operation lifetimes of OLEDs with different host materials were measured at an initial luminance of 500 cd m^−2^. The decrease in luminance with increasing OLED operation time was measured using the LT50 parameter, which represents the time at which the luminance decreased to 50% of the initial luminance. Figure 4e shows the normalized luminance of the OLEDs as a function of OLED operation time under a constant current density at the initial luminance of 500 cd m^−2^. Consistently with the previous report in which mCBP‐CN was suggested as a stable host,^[^ ^19^ ^]^ the LT50 of the OLED fabricated using mCBP‐CN (36.2 h) was much longer than that of the DPEPO‐based OLED (0.6 h) and the conventional mCBP‐based OLED (3.3 h). Nevertheless, the H2‐, H3‐, and H4‐based OLEDs notably exhibit much longer operation lifetimes than not only the DPEPO‐ and mCBP‐based OLEDs, but also the mCBP‐CN‐based OLED. In particular, the H3‐based OLED exhibited an operation lifetime comparable to that of the stable mCBP‐CN‐based OLED, while simultaneously exhibiting remarkably improved efficiency (Figure 4c). Furthermore, the H2‐ and H4‐based OLEDs exhibited much longer LT50 (122.4 and 86.9 h, respectively) than the mCBP‐CN‐based OLED. While the mCBP‐CN‐based OLED had the best charge transport characteristics as shown in Figure 4b, and the most suppressed efficiency roll‐off, as shown in Figure 4c, indicating the best charge balancing ability, its EQE at a practical brightness and LT50 were inferior to those of the OLEDs based on H2–H4.

As we discussed with Figure 1, the simultaneously high efficiency and OLED operation stability are attributed to the high polarity of the novel hosts, stabilizing CT excited states of 1PCTrz, and the phosphine oxide‐free molecular structure with strong electron‐transporting character, which enhances operation stability. However, the LT50 of the H1‐based OLED was measured shorter than that of the mCBP‐CN‐based OLED (21.7 vs 36.2 h), while its EQE was superior. This exception can be explained by considering the charge transporting character of H1. Owing to the CN‐functionalization, the four novel host materials (H1–H4) all have low‐lying HOMO and LUMO levels (Figure 1b), which provides electron‐transporting characteristics and high device stability with highly doped (20 vol% here) hole‐transporting TADF emitters.^[^ ^19^ ^]^ With this knowledge, the shorter LT50 of the H1‐based OLED can be explained by considering the shallower LUMO energy level of H1, which indicates weaker electron‐transporting character. This explanation is valid when comparing the device operation lifetimes of the OLEDs based on the high‐polarity host materials with the same core structures (H1 and H2; H3 and H4). The H2‐based TADF device exhibits a much longer device operation lifetime than the H1‐based device (122.4 vs 21.7 h), probably because H2 has a lower LUMO than H1 (−2.04 vs −1.75 eV). Meanwhile, the H4‐based TADF device exhibits a longer device operation lifetime than H3‐based device (86.9 vs 55.1 h) for the same reason; the LUMO energy levels of H4 and H3 are −1.79 and −1.64 eV, respectively. Through those comparisons, we can conclude that host materials with lower LUMO are more suitable for enhancing device stability of TADF devices that employ highly doped hole‐transporting TADF emitters. Almost the same results with the characteristics of OLEDs employing other blue TICT‐type TADF emitters were achieved, which confirms the versatility of our new host materials. One of the examples employing recently reported blue TICT TADF emitter, 9‐(5′‐(4,6‐diphenyl‐1,3,5‐triazin‐2‐yl)‐[1,1′:3′,1′′‐terphenyl]‐2′‐yl)‐3,6‐diphenyl‐9H‐carbazole (PPCzTrz),^[^ ^34^ ^]^ is presented in Figure S6, Supporting Information.

### 2.5 Characteristics and Stability of Hole‐Only Devices and Electron‐Only Devices

**Figure 5:**
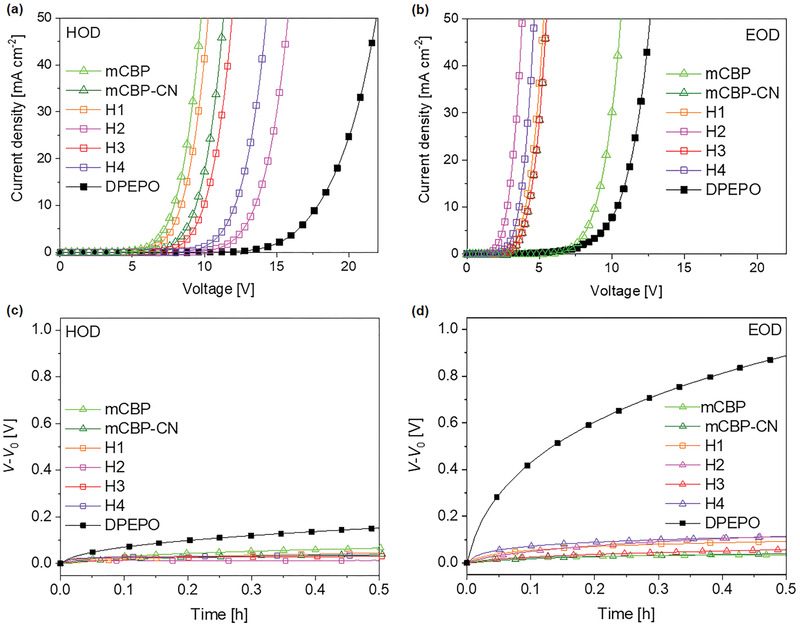
Characteristics of hole‐only devices (HODs) and electron‐only devices (EODs). a,b) Current density–voltage curves of HODs and EODs, respectively, for various host films. c,d) Changes of the driving voltages of HODs and EODs, respectively, for various host films as a function of operation time with constant driving current. *V*_0_ is the initial voltage.

To investigate the electrical stability of the novel hosts and to explain the longer operation lifetimes of the OLEDs employing the hosts, we performed charge transport stability test of the host films in hole‐only devices (HODs) and electron‐only devices (EODs). **Figure** **5**a,b shows the current density–voltage (*J*–*V*) characteristics of the HODs and EODs, respectively. The EODs with the high‐polarity hosts had much lower turn‐on voltages than the HODs, indicating that they are electron‐transporting hosts, similar to mCBP‐CN. Electron‐transporting hosts are well‐suited to use with hole‐transporting TADF emitters with a shallow HOMO and high doping concentration, which facilitates the fabrication of stable OLEDs.^[^ ^19^ ^]^ Figure 5c,d shows the increment of the driving voltages (*V*_d_) of the HODs and EODs, respectively, under the same current density (50 mA cm^−2^) as a function of time. The increase of *V*_d_ is equivalent to the increase of resistivity induced by material degradation.^[^ ^35^ ^]^ For all the novel high‐polarity hosts (H1–H4), the HODs and EODs showed much smaller increments in *V*_d_ during operation than the DPEPO‐based HOD and EOD. This indicates that the novel hosts will degrade much slower than DPEPO under the same electrical stress, in the same manner as mCBP‐CN although they can provide high polarities which are comparable to DPEPO.

### 2.6 Photoluminescence Stability

**Figure 6:**
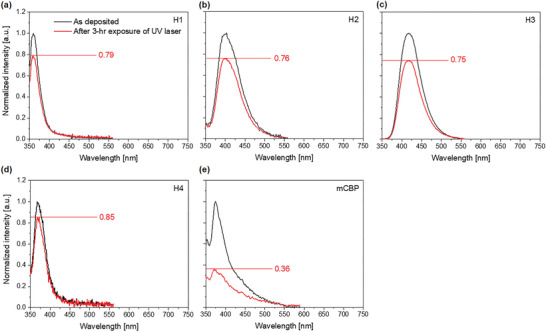
PL stability of various host films. PL spectra of the a) H1, b) H2, c) H3, d) H4, and e) mCBP films. The black PL spectra were achieved from the as‐deposited films while the red PL spectra were achieved from the very films after 3‐h exposure to UV laser.

To investigate and compare the excited‐state stabilities of the hosts, we performed a PL‐stability study by exposing the films to a UV laser for 3 h. **Figure** **6** shows normalized PL spectra of 50‐nm high dipole moment H1–H4 and mCBP films in the as‐deposited (black) and degraded (red) conditions. Normalization was performed by dividing the spectra by the PL peak intensity of the as‐deposited film. The peak intensities for the H1–H4 films decreased to at most 75% of its initial value after 3 h exposure to UV‐laser, while that of mCBP film decreased to 36%. This significant difference in PL stability correlates with the great difference in device operation lifetime between the novel hosts (H1–H4)‐ and mCBP‐based OLEDs (Table 2). The high PL stability of the mCBP‐CN film has been already presented in the previous report.^[^ ^19^ ^]^ We did not perform PL stability studies on DPEPO because it requires even higher energy laser for excitation. However, a few articles have reported low photochemical stability of the phosphine oxide moiety,^[^ ^17^, ^18^ ^]^ and we have previously discussed the poor PL stability of DPEPO and its substitutions with two phosphine oxide moieties.^[^ ^19^ ^]^

### 2.7 Orientation of Transition Dipole Moments

**Figure 7:**
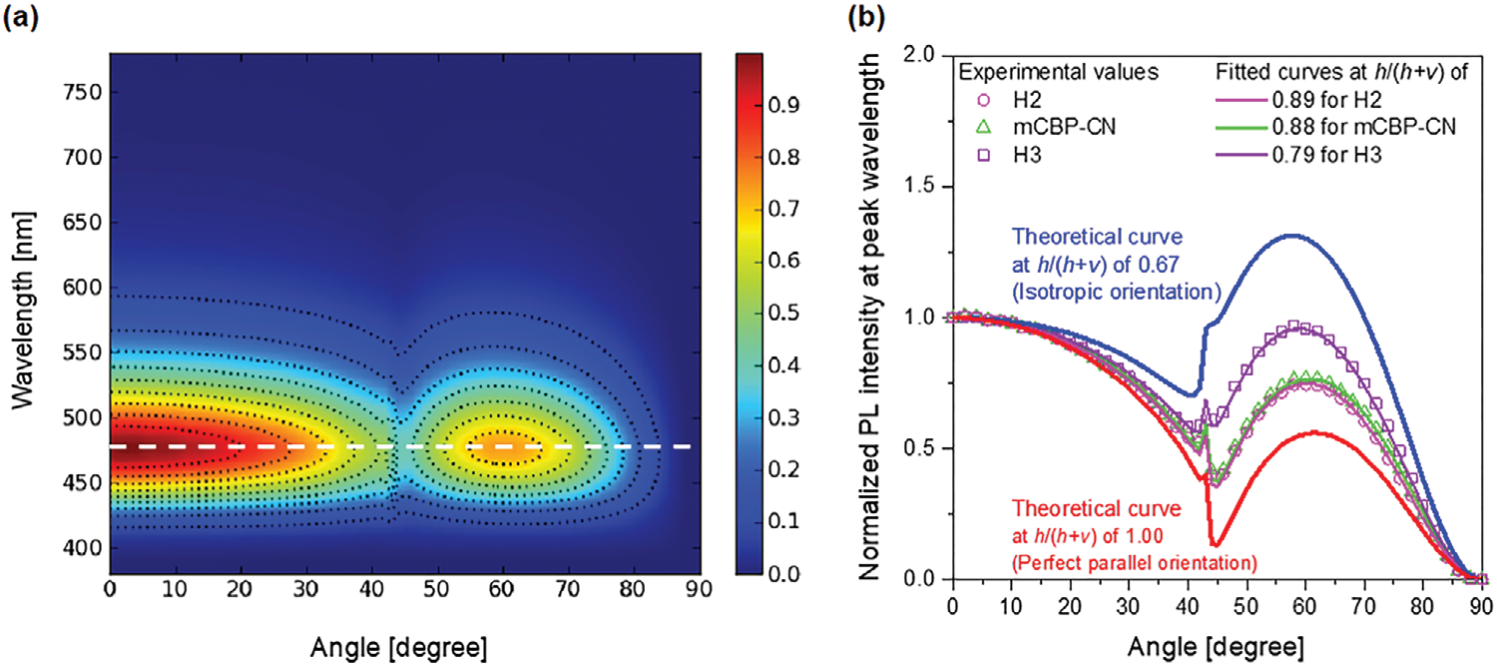
Analysis of the horizontal orientations of the transition dipole moments (*h*/(*h* + *v*)). a) Angle‐dependent PL spectrum of *p*‐polarized light from a 50‐nm‐thick H2:1PCTrz (85:15 by volume) film. b) Angle‐dependent PL intensities at peak wavelengths. The magenta open squares represent the PL intensity from the H2:1PCTrz film, which are equivalent to the white dashed line in (a). The green triangles and purple squares represent the PL intensities from mCBP:1PCTrz (85:15) and H3:1PCTrz (85:15) films, respectively. Inspection of the solid curves for the films of the three 15% 1PCTrz‐doped hosts (H2, mCBP‐CN, and H3) reveal horizontal orientations of the transition dipole moment (*h*/(*h* + *v*)) of 0.89, 0.88, and 0.79, respectively. The blue and red lines correspond to theoretical curves constructed with *h*/(*h* + *ν*) values of 0.67 (isotropic orientation) and 1.0 (perfect parallel orientation), respectively. See also Figure S8, Supporting Information.

To further verify the mechanism of host polarity‐induced efficiency enhancement, we performed angle‐dependent PL measurements with various host films doped with 1PCTrz, which enables the cause of the improved EQE_max_ to be more closely examined. In particular, we can observe whether the efficiency enhancement in this work originates from light out‐coupling effects^[^ ^36^ ^]^ more dominantly than host polarity or not. **Figure** **7**a shows the angle‐dependent PL spectrum of *p*‐polarized light from the 50‐nm‐thick 1PCTrz‐doped H2 host film, while the angle‐dependent PL intensities for H2:1PCTrz are depicted in Figure 7b (magenta squares). A theoretical fit of the data reveals a horizontal orientation of the transition dipole moments (*h*/(*h* + *ν*)) of 0.89. This value is much higher than that for an isotropic orientation (*h*/(*h* + *ν*) = 0.67), which indicates that the orientation effect is significant. However, the mCBP‐CN:1PCTrz film also exhibits a comparable *h*/(*h* + *ν*) value of 0.88. This means that the significant difference in EQE_max_ between the H2‐ and mCBP‐CN‐based OLEDs is not attributed to orientation effects. Moreover, in spite of the small *h*/(*h* + *ν*) of 0.79 of the H3:1PCTrz emitting layer, which indicates low out‐coupling efficiency, the OLED with the H3:1PCTrz emitting layer exhibited the highest EQE_max_ among the OLEDs with phosphine oxide‐free hosts. For the tested films, there were no significant differences in the *h*/(*h* + *ν*) values, with the exception of the H3:1PCTrz film. This indicates that the enhanced EQE_max_ observed with high‐polarity host‐based TADF OLEDs is not attributed to the orientation effect. Considering the results of the PL spectrospcopic study, including the redshifted and broadened emission peaks and higher delayed fluorescence quantum yields with high‐polarity host‐based films, we conclude that the enhanced efficiencies of the TADF devices are ascribed to the stabilization of the CT excited states of the TADF emitter when surrounded by high‐polarity host molecules. The values of *h*/(*h* + *ν*) for all the tested films are summarized in **Table** **3** along with their expected out‐coupling efficiencies. The angle‐dependent PL intensities for the other hosts and their theoretical fit are presented in the Figure S8, Supporting Information.

**Table 3:** Out‐coupling efficiency applying horizontal orientation of the transition dipole moment

	*h*/(*h* + *ν*)^a)^	*η*_o.c._ ^b)^
mCBP:1PCTrz	0.83	0.248
mCBP‐CN:1PCTrz	0.88	0.263
H1:1PCTrz	0.87	0.260
H2:1PCTrz	0.89	0.266
H3:1PCTrz	0.79	0.236
H3:1PCTrz	0.88	0.263
DPEPO:1PCTrz	0.87	0.260

^a)^ Horizontal orientation of transition dipole moment;

^b)^ Out‐coupling efficiency applying *h*/(*h* + *ν*). In case of the perfect isotropic orientation, *η*_o.c._ = 0.2.

## 3 Conclusion

We have designed, synthesized, and verified four new phosphine oxide‐free host materials, H1–H4, for using with a blue TADF emitter molecule, 1PCTrz. The host materials had molecular asymmetry and large electric dipole moments. The TADF devices based on the novel hosts exhibited high efficiencies approaching that of the DPEPO‐based TADF device. At the same time, they achieved much longer operation lifetimes than those based on DPEPO, mCBP, and mCBP‐CN. This simultaneous improvements in efficiency and stability are attributed to the intricate properties of the host materials that works consonantly toward enhanced device operation: i) high polarity stabilizes the CT excited state of the TADF emitter; ii) a relatively low magnitude of excited‐state dipole moment suppresses intermolecular CT‐induced quenching of excitons; iii) strengthened electron‐transporting character allows mixed‐host‐like behavior in the emitting layer owing to the hole‐transporting ability of the highly doped TADF emitter; and iv) phosphine oxide‐free design improves photochemical stability. Our novel host materials adopt asymmetric molecular structures and polar groups appropriately to cope with the trade‐off between high ground‐state dipole moments and low excited‐state dipole moments, while simultaneously modulating their molecular orbital energy levels. Our strategy for designing hosts with particular characteristics will promote the commercialization and success of TADF devices in OLED technology.

## 4 Experimental Section

### Calculations of Dipole Moment

The molecular structures of the phosphine oxide‐free hosts considered in this study had rotatable bonds and thus allowed various conformations, which might give a wide range of dipole moments. It was expected that variations of the dipole moment may affect the polarity of the host matrix. In order to find an appropriate set of conformers, a conformational search module included in Schrödinger software^[^ ^37^ ^]^ that collects structures using the mixed torsional/low‐mode sampling method starting from a given structure was used. A set of representative conformers were determined from the local minimization starting from numerous structures. In the minimization, the energies were evaluated in the classical level using the OPLS3e force field. For all conformers, optimizations were performed through density functional theory calculations at the level of B3LYP/def2‐SVP and obtained the values of dipole moments using the TURBOMOLE 7.5 program.^[^ ^38^ ^]^ The representative value of the dipole moment, denoted by *μ*_GS_, was obtained from the lowest energy structure with the minimum energy among the conformers. The minimum and the maximum values of the dipole moments in company with *μ*_GS_ for all hosts are displayed in Figure S9, Supporting Information. The phosphine oxide‐free hosts exhibited a considerable variation of dipole moments according to conformations. *μ*_GS_ was regarded as a polarity scale for convenience in this study; however, one may consider the range of dipole moments due to the conformer distributions of the hosts, especially when the hosts may have similar *μ*_GS_ but distinguished polarities. The excited‐state dipole moments were calculated at the spin‐component‐scaled‐CC2 level of the second‐order approximate coupled‐cluster singles and doubles (CC2) calculations^[^ ^39^ ^]^ with the first‐excited singlet state geometries which were optimized by the TD‐B3LYP level without any symmetry constraints. The resolution‐of‐the‐identity approximation was used in all calculations.

### General Procedures of Synthesis and Characterization

Chemicals were purchased from Sigma Aldrich Co., Tokyo Chemical Industry Co., 4chem Laboratory Co., Ltd., Medigen Co. Ltd., and Hanchem Co., Ltd. and used without further purification. ^1^HNMR and ^13^CNMR spectra were recorded on a Bruker ASCEND 500 at 500 MHz using CD_2_Cl_2_ as the solvent. The ion trap time‐of‐flight liquid chromatograph mass spectrometer (LCMS‐IT‐TOF) system instrument consisted of LC‐30A Nexera SR system instrument (Shimadzu) connected to a hybrid IT‐TOF mass spectrometer equipped with an electrospray ionization source (Shimadzu). The UV–vis spectra were obtained by means of a Varian model UV–vis–NIR spectrophotometer 5000 and the fluorescence spectra were measured on a HITACHI F7000 spectrometer for the solution states. The UV–vis absorption and solution PL emission spectra of host materials were obtained from dilute tetrahydrofuran, toluene, and dimethyl sulfoxide solutions (1 × 10^−5^ m), while the triplet energy values of the host materials were obtained from the PL spectra at 77 K using liquid nitrogen. The details of the synthesis of the hosts and precursors are presented in Supporting Information.

### Measurement of HPLC Retention Time

The HPLC retention time was measured with a Shimadzu LC‐30A Nexera SR System equipped with a diode array detector and reversed‐phase type ACQUITY CSH C‐18 2.1 × 100 mm, 1.7 µm chromatographic columns. The measurement conditions were set as follows: acetonitrile and water for mobile phase solvents, 0.5 mL min^−1^ flow rate, and tetrahydrofuran for sampling solvent.

### Measurement of PL Characteristics of Thin Films

The transient PL decay characteristics were measured at room temperature under a nitrogen atmosphere using a fluorescence spectrometer (PicoQuant, FluoTime 300) based on time‐correlated single photon counting (PicoQuant, PicoHarp 300). A pulsed LED (PicoQuant, PLS 340) with an excitation wavelength of 340 nm and a single photon sensitive photomultiplier tube (PicoQuant, PMA‐C) were used. PLQY was measured at room temperature under a nitrogen atmosphere using an absolute PLQY measurement system (Quantaurus‐QY, Hamamatsu). The PL stability test and comparison between the as‐deposited and 3‐h UV‐laser‐exposed films was performed using a He‐Cd laser (KIMMON KOHA, IK3202R‐D) at 3.5 mW with an excitation wavelength of 325 nm. The films for the PL stability test were glass‐encapsulated in a nitrogen‐filled glove box after vacuum deposition. The angle‐dependent PL was measured using a continuous wave laser (325 nm, Melles Griot). The incident angle of the excitation source was fixed at 45°. The angle dependent PL spectra of P‐polarized light were detected using charge‐coupled device (MAYA 2000, Ocean Optics).

### Device Fabrication and Measurement

The organic layers were deposited on pre‐cleaned ITO glass substrates using a thermal evaporation system with a vacuum pressure of <1.0 × 10^−6^ torr. Layers of Liq (1 nm thick) and Al (100 nm thick) were deposited by thermal evaporation to form the cathode. The deposition rates of the organic and metal layers were about 0.1 and 0.5 nm s^−1^, respectively, while that of the Liq layer was about 0.01 nm s^−2^. The active device area of 4 mm^2^ was defined by the overlapped area of the ITO and Al electrodes. The HOD structure was ITO/p‐doped BCFA (3 wt%, 10 nm)/BCFA (135 nm)/host(30 nm)/BCFA (10 nm)/Al. The EOD structure was ITO/Ag/DBFPO:Liq (10 nm)/test material (30 nm)/DBFPO:Liq (30 nm)/Liq (1 nm)/Ag. The current, voltage, and luminance of the OLEDs were measured using a Keithley 2400 Source‐Meter and Topcon SR‐3AR spectroradiometer. Lifespan measurements of the OLEDs were performed under constant current.

## Conflict of Interest

The authors declare no conflict of interest.

## Supporting information

Supporting Information

## Data Availability

Research data are not shared.
