# Supplementary material for: Dipole Moment‐ and Molecular Orbital‐Engineered Phosphine Oxide‐Free Host Materials for Efficient and Stable Blue Thermally Activated Delayed Fluorescence
Source: Adv Sci (Weinh). 2021 Nov 21;9(3):2102141. doi: 10.1002/advs.202102141 (PMC8787426; doi:10.1002/advs.202102141)
Supplement: Supplementary file 1 — Supporting Information [file ADVS-9-2102141-s001.pdf]

WILEY-VCH

© 2021 Wiley-VCH GmbH

ADVANCED SCIENCE
Open Access

Supporting Information

for *Adv. Sci.,* DOI: 10.1002/advs.202102141

Dipole Moment- and Molecular Orbital-Engineered Phosphine Oxide-Free Host Materials for Efficient and Stable Blue Thermally Activated Delayed Fluorescence

Soo-Ghang Ihn, Daun Jeong, Eun Suk Kwon, Sangmo Kim, Yeon Sook Chung, Myungsun Sim, Jun Chwae, Yasushi Koishikawa, Soon Ok Jeon, Jong Soo Kim, Joonghyuk Kim, Sungho Nam, Inkoo Kim, Sangho Park, Dae Sin Kim, Hyeonho Choi, and Sunghan Kim*

Supporting Information

Dipole Moment- and Molecular Orbital-Engineered Phosphine Oxide-Free Host Materials for Efficient and Stable Blue Thermally Activated Delayed Fluorescence

Soo-Ghang Ihn, Daun Jeong, Eun Suk Kwon, Sangmo Kim, Yeon Sook Chung, Myungsun Sim, Jun Chwae, Yasushi Koishikawa, Soon Ok Jeon, Jong Soo Kim, Joonghyuk Kim, Sungho Nam, Inkoo Kim, Hyeonho Choi, and Sunghan Kim*

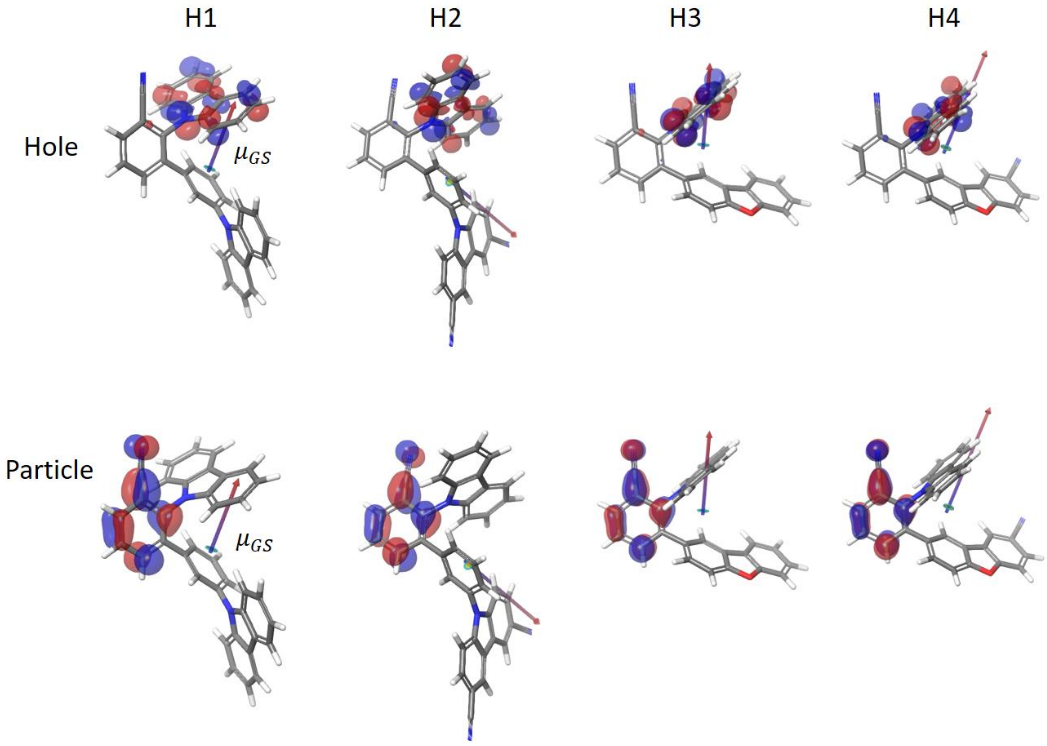

Figure S1. Natural transition orbitals (NTOs) in the charge-transfer excited state of H1-H4. The directions and magnitudes of the ground-state dipole moments μ_{GS} are also depicted with arrows.

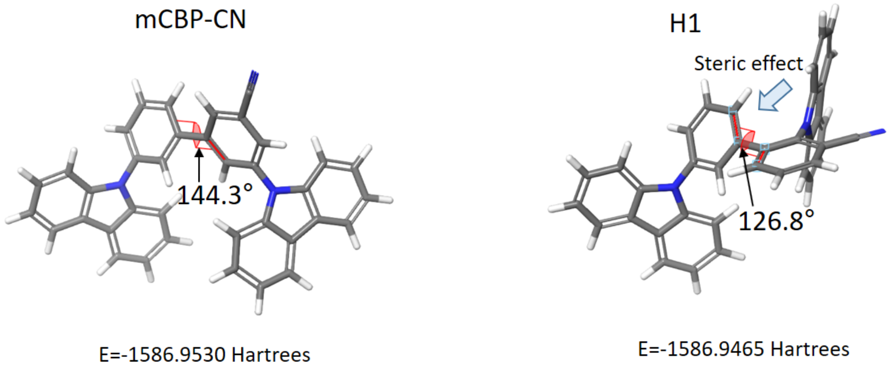

Figure S2. Torsonal angles regarding the bond connecting phenyl and CN-substituted phenyl for **(a)** mCBP-CN **(b)** H1.

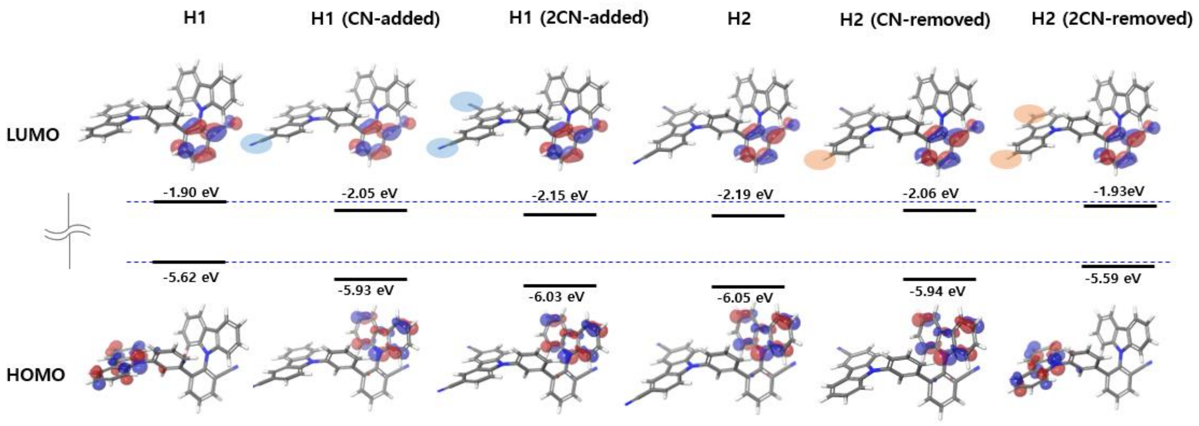

Figure S3. The energy levels and locations of HOMO and LUMO with the addition (removal) of the electron withdrawing groups, -CN, to the optimized geometry of H1 (from that of H2) obtained via single-point DFT calculations.

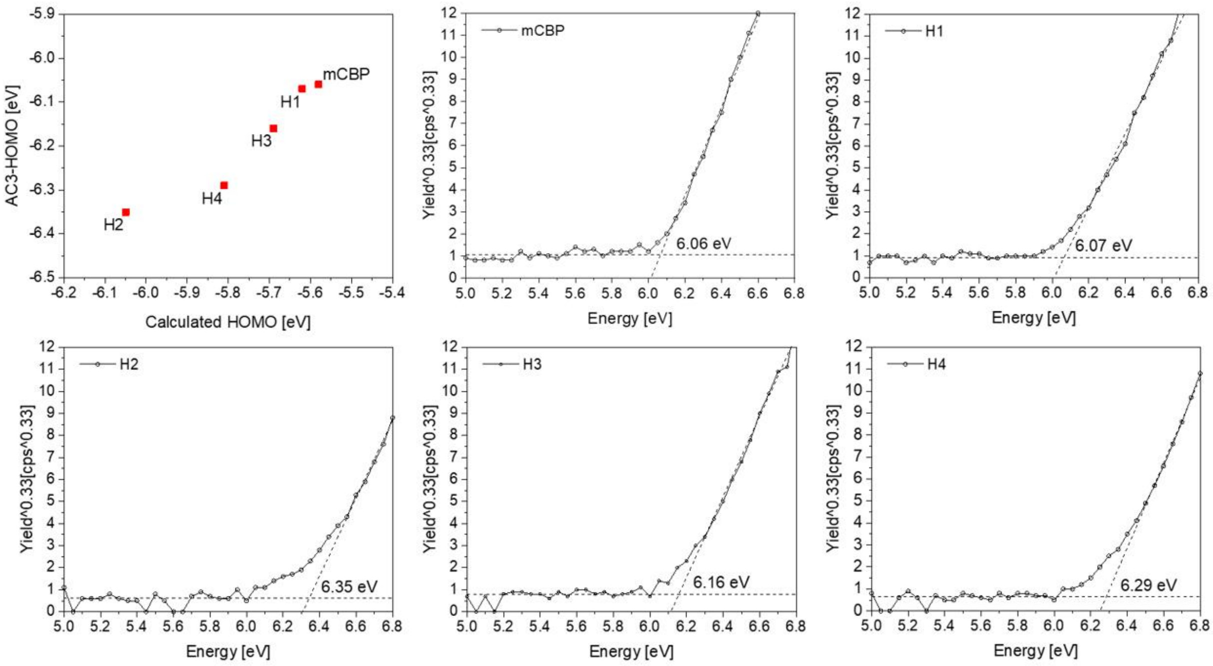

Figure S4. Comparison between calculated and measured HOMO energy levels of H1–H4 and the results of the photoelectron spectroscopy in air (PESA) measurements performed on AC-3 (RIKEN KEIKI Co., LTD.).

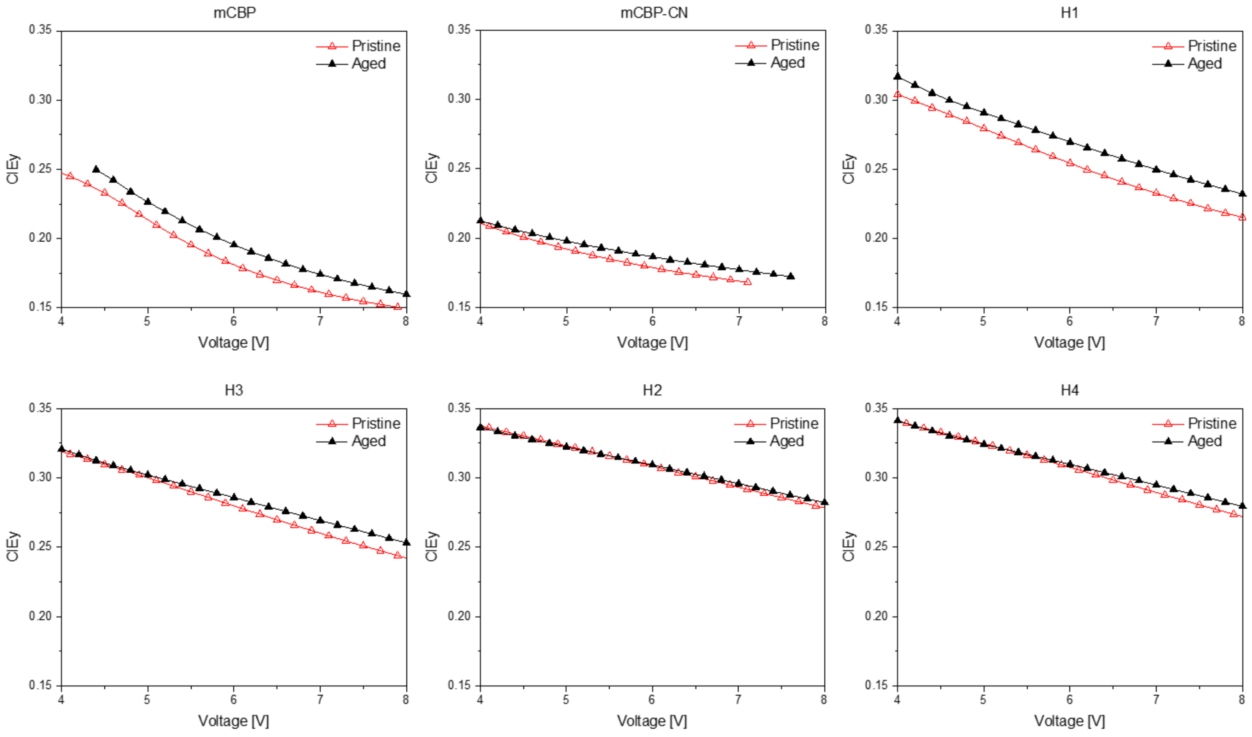

Figure S5. EL spectrum shift prisitine and aged EL device operation. Increment of CIEy value after aging (EL device operation) is observed for each host material, which indicates redshift induced by the materials degradation-mediated shifts of recombination sites. Higher CIEy values indicating redshift induced by high-polarity hosts (H1 – H4) than those of mCBP and mCBP-CN-based devices are also observed for both pristine and aged devices.

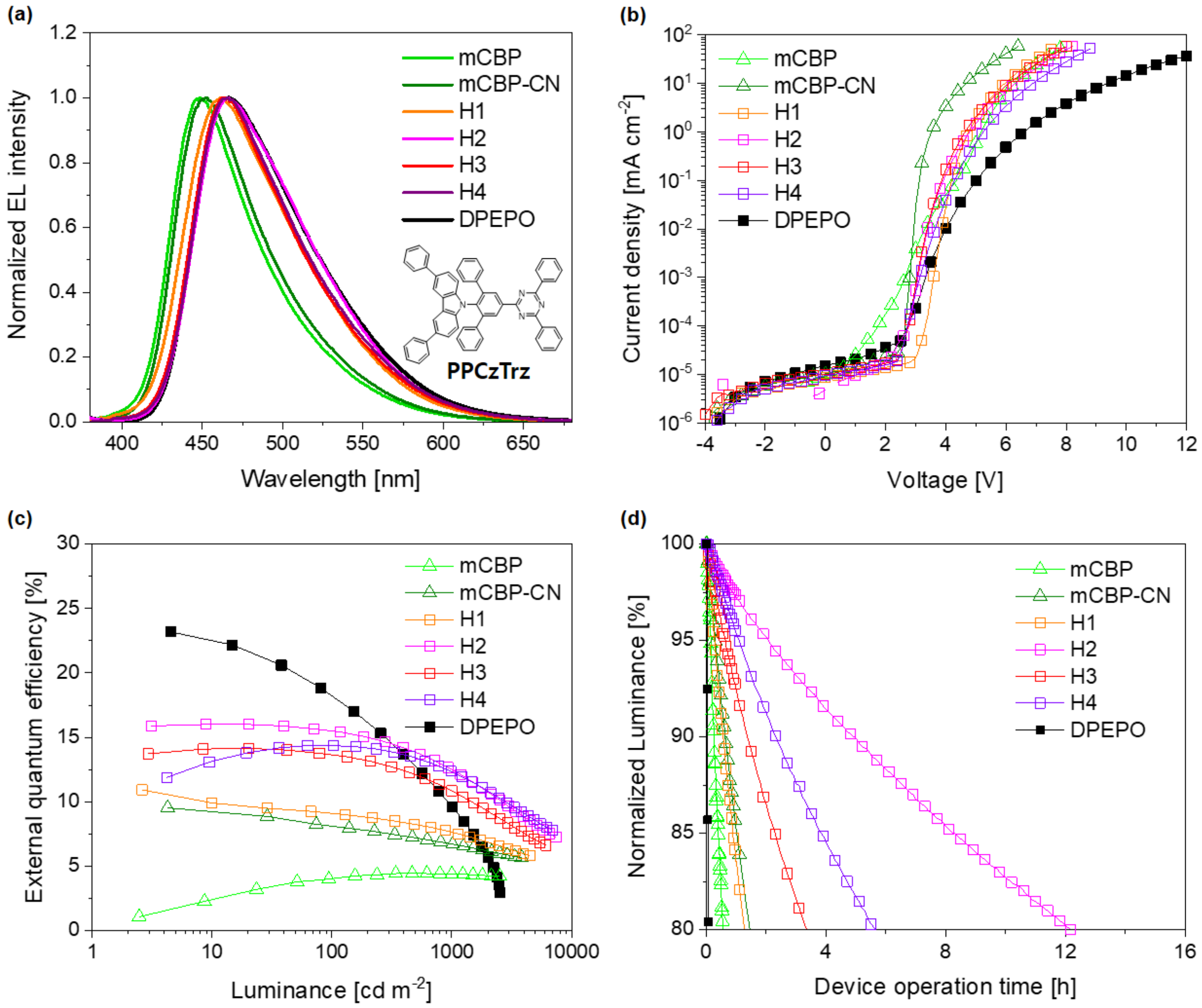

Figure S6. Characteristics of OLEDs employing 9-(5′-(4,6-diphenyl-1,3,5-triazin-2-yl)-[1,1′:3′,1′′-terphenyl]-2′-yl)-3,6-diphenyl-*9H*-carbazole (PPCzTrz). **(a)** Electroluminescence spectra of the OLEDs at 500 cd m^{-2}. The insert is the chemical structure of PPCzTrz. **(b)** Current density–voltage characteristics for the OLEDs. **(c)** EQE of the OLEDs as a function of)luminance. **(d)** Normalized luminance of the OLEDs as a function of operation time at a constant current density. The initial luminance was 500 cd m^{-2}.

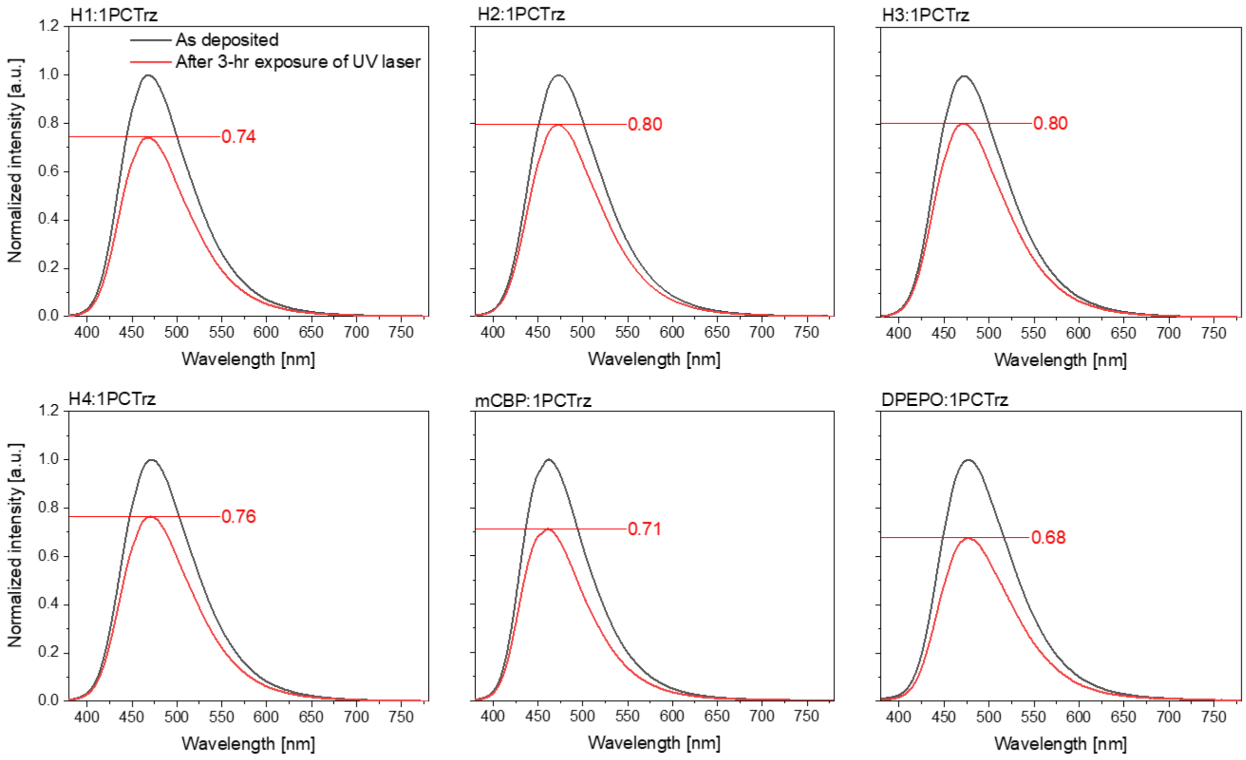

Figure S7. PL stability of various host films with 1PCTrz doping.

We performed PL stability studies with 1PCTrz-doped host films, which enables the excited-state stability to be investigated without detrimental host interactions such as concentration quenching, triplet-triplet annihilation, exciton migration, etc.[1-5] Figure S7 show normalized PL spectra of 50-nm 1PCTrz-doped hosts films. The peak intensity for the high-dipole moment host (H1–4):1PCTrz films decreased to at most 74% of their initial values after 3h UV exposure, while those for the mCBP:1PCTrz and DPEPO:1PCTrz films decreased to 71% and 68%, respectively. This result also explains the longer operation lifetime of the high-dipole moment host (H1–4):1PCTrz-based OLEDs, although the decrement of the peak intensity for mCBP:1PCTrz and DPEPO:1PCTrz films was reduced when compared to that for the host-only films. The improved PL stability after 1PCTrz doping is attributed to the host-to-guest energy transfer for mCBP. However, for DPEPO, the unexpectedly low loss of intensity after 1PCTrz doping is related to the fact that DPEPO was

not excited during the DPEPO:1PCTrz film PL measurement, so it did not suffer the degradation induced by the phosphine oxide-substituted moiety.[6,7]

References

[1] T. Chatterjee, K.-T. Wong, *Adv. Opt. Mater.* **2019**, *7*, 1800565.

[2] Z. Yang, Z. Mao, Z. Xie, Y. Zhang, S. Liu, J. Zhao, J. Xu, Z. Chi, M. P. Aldred, *Soc. Rev.* **2017**, *46*, 915-1016.

[3] J. H. Park, J. H. Seo, S. H. Lim, G. Y. Ryu, D. M. Shin, Y. K. Kim, *J. Phys. Chem. Solids* **2008**, *69*, 1314-1319.

[4] C.-T. Chen, *Chem. Mater.* **2004**, *16*, 4389-4400.

[5] P. E. Burrows, S. R. Forrest, S. P. Sibley, M. E. Thompson, *Appl. Phys. Lett.* **1996**, *69*, 2959-2961.

[6] N. Lin, J. Qiao, L. Duan, H. Li, L. Wang, Y. Qiu, *J. Phys. Chem. C.* **2012**, *116*, 19451.

[7] N. Lin, J. Qiao, L. Duan. L. Wang, Y. Qiu, *J. Phys. Chem. C* **2014**, *118*, 7569.

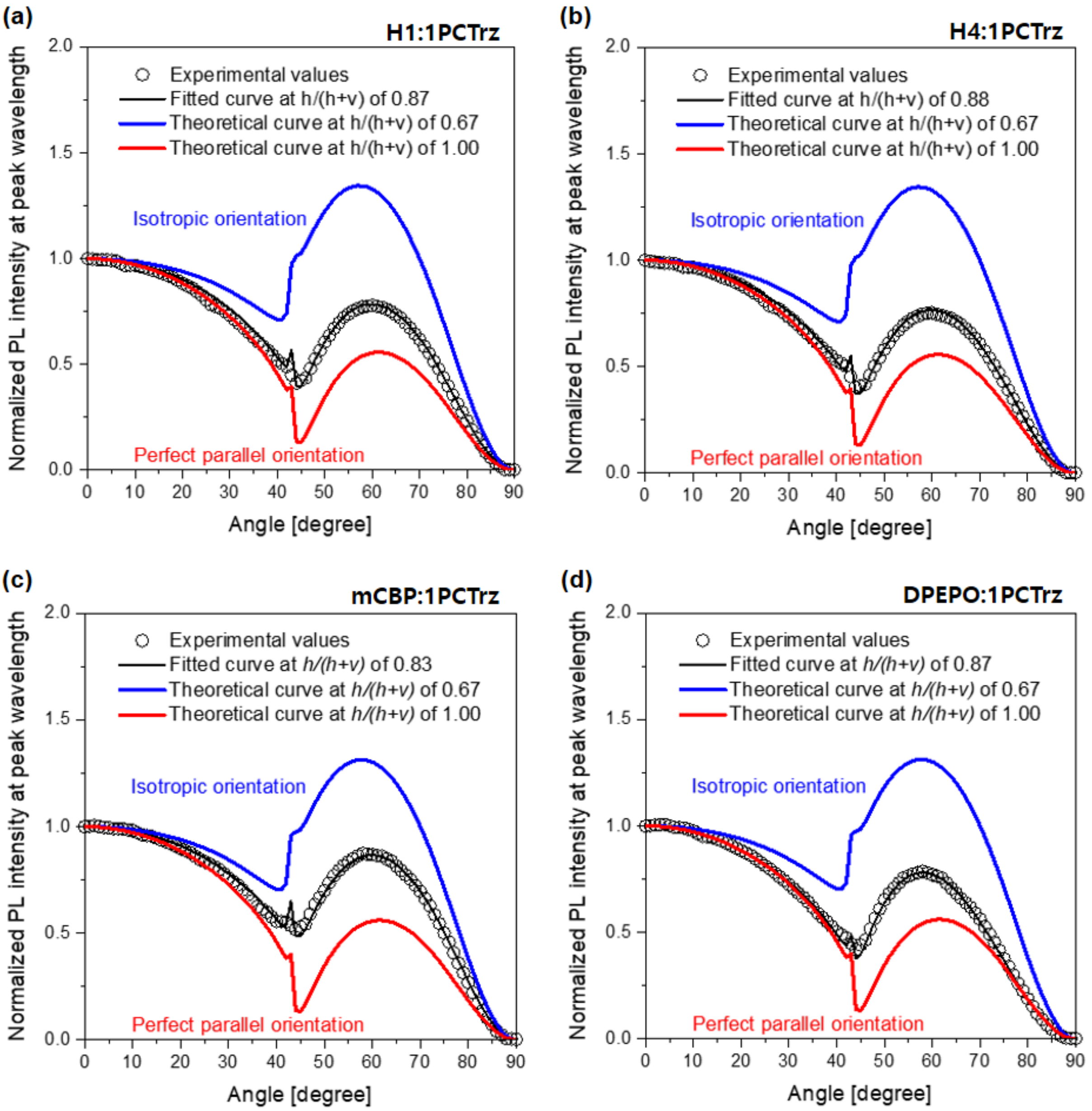

Figure S8. Analysis of the horizontal orientations of the transition dipole moments, $h/(h+v)$, , related Figure 7. Angle-dependent PL intensities at peak wavelengths. The black open squares represent the PL intensities from **(a)** H1:1PCTrz, **(b)** H4:1PCTrz, **(c)** mCBP:1PCTrz, and **(d)** DPEPO:1PCTrz films. Inspection of the solid curves for the films of the four 15% TADF emitter (1PCTrz)-doped hosts (H1, H4, mCBP, and DPEPO) reveal that the horizontal orientations of ($h/(h+v)$) are 0.87, 0.88, 0.83, and 0.87, respectively. The blue and red lines correspond to theoretical curves constructed with $h/(h+v)$ values of 0.67 (isotropic orientation) and 1.0 (perfect parallel orientation), respectively.

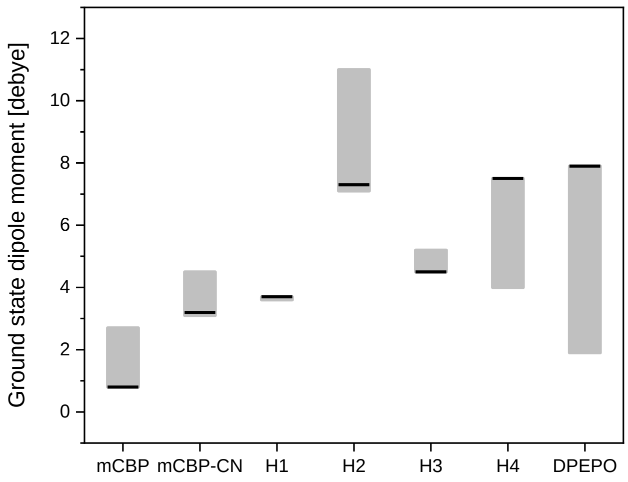

Figure S9. Ground-state dipole moments μ_{GS} of hosts calculated from various conformer structures. The representative value, corresponding to the minimum energy structure for each host, is marked with a black solid line.

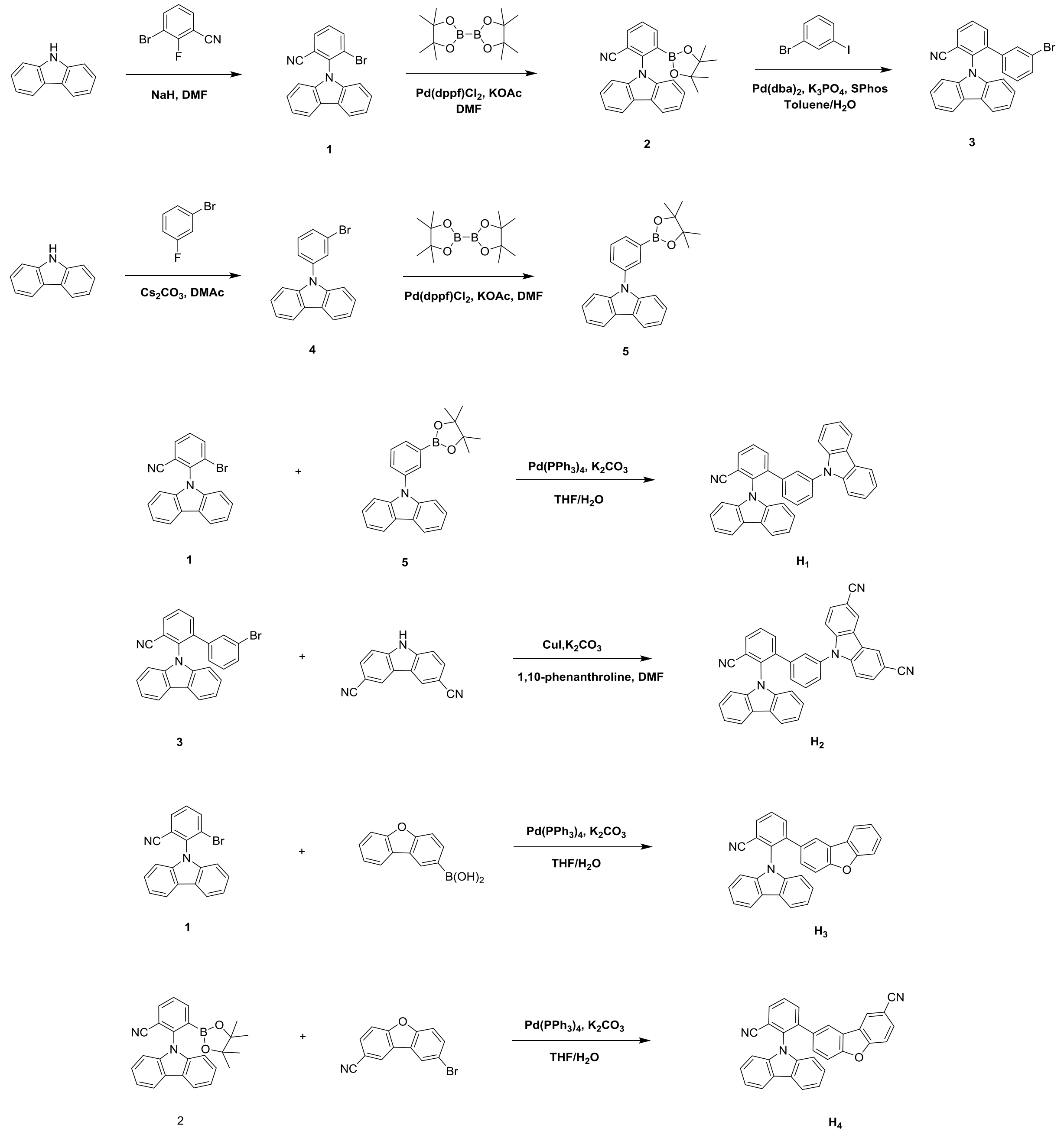

Figure S10. Synthetic route for H1, H2, H3, and H4.

Synthesis of H1: A mixture of 3-bromo-2-(9*H*-carbazol-9-yl)benzonitrile (**1**) (6.81 g, 19.62 mmol), 9-(3-(4,4,5,5-tetramethyl-1,3,2-dioxaborolan-2-yl)phenyl)-9*H*-carbazole (**5**) (15.12 g, 39.25 mmol), potassium carbonate (5.42 g, 39.25 mmol), and tetrakis(triphenylphosphine) palladium(**0**) (2.27 g, 1.96 mmol) in 70 mL of THF/water (2.5:1) was stirred at 85 °C for 12 h. After allowing it to cool to room temperature, the reaction mixture was diluted with methanol

and filtered. The resulting solid was purified by column chromatography using dichloromethane/*n*-hexane (1:2) as eluent. The white solid obtained after evaporating the solvent was recrystallized from ethyl acetate and finally dried under vacuum to give *2,3′-di(9H-carbazol-9-yl)-[1,1′-biphenyl]-3-carbonitrile* (**H1**) (5.40 g, 54%). The synthetic routes for the hosts are displayed in Figure S10. ^{1}**H NMR** (500 MHz, CD_2Cl_2): δ 8.13 (d, J = 7.7 Hz, 2H), 8.06 (dd, J = 7.7, 0.4 Hz, 2H), 7.96 (d, J = 7.7 Hz, 2H), 7.78 (t, J = 7.8 Hz, 1H), 7.38 (t, J = 8.0 Hz, 2H), 7.21 – 7.31 (m, 9H overlap), 7.08 (d, J = 8.2 Hz, 2H), 7.09 (d, J = 2.7 Hz, 1H), 6.79 (d, J = 8.2 Hz, 2H); ^{13}**C NMR** (126 MHz, CD_2Cl_2): δ 143.23, 141.3, 141.2, 139.4, 138.2, 138.0, 136.7, 134.5, 130.4, 130.3, 127.6, 124.2, 126.8, 126.4, 124.1, 123.6, 121.2, 121.2, 120.6, 120.4, 116.4, 115.5, 110.3, 109.9; **LCMS-IT-TOF**: 510.19 [$(M+H)^+$]. See Figure S11 and S12.

Synthesis of H2: A mixture of 3′-bromo-2-(9*H*-carbazol-9-yl)-[1,1′-biphenyl]-3-carbonitrile (**3**) (5.48 g, 12.06 mmol), 9*H*-carbazole-3,6-dicarbonitrile (3.93 g, 18.09 mmol), potassium carbonate (5.00 g, 36.19 mmol), 1,10-phenanthroline (4.35 g, 24.12 mmol), and copper iodide (2.30 g, 12.06 mmol) in 30 mL of dimethylacetamide was stirred at 170 °C for 15 h. After allowing it to cool to room temperature, the reaction mixture was diluted with methanol,filtered, and dried. The resulting solid was diluted with DMF and filtered with silica gel. The yellow solid obtained after evaporating the solvent was recrystallized from 1,4-dioxane and finally dried under vacuum to give 9-(2′-(9*H*-carbazol-9-yl)-3′-cyano-[1,1′-biphenyl]-3-yl)-9*H*-carbazole-3,6-dicarbonitrile (**H2**) (5.40 g, 47%). ^{1}**H NMR** (500 MHz, CD_2Cl_2): δ 8.38 (d, J = 1.1Hz, 2H), 8.08 (d, J = 7.7 Hz, 2H), 7.99 (td, J = 7.7, 1.5 Hz, 2H), 7.81 (t, J = 7.8 Hz, 2H), 7.59 (dd, J = 8.6, 1.5 Hz, 2H), 7.31 – 7.37 (m, 4H overlap), 7.17 (dt, J = 6.2, 1.7 Hz, 1H), 7.01 – 7.04 (m, 3H overlap), 6.65 (d, J = 7.6 Hz, 2H); ^{13}**C NMR** (126 MHz, CD_2Cl_2): δ 143.6, 142.6, 141.2, 139.9, 137.9, 136.5, 135.7, 134.9, 131.1, 130.9, 130.5,

129.6, 127.7, 127.0, 126.9, 126.0, 124.0, 122.7, 121.3, 121.1, 121.1, 120.1, 116.2, 115.7, 111.4, 110.2, 104.7; **LCMS-IT-TOF**: 560.17 [$(M+H)^+$]. See Figure S13 and S14.

Synthesis of H3: 2-(9*H*-Carbazol-9-yl)-3-(dibenzo[*b*,*d*]furan-2-yl)benzonitrile (**H3**) was obtained as white crystal (13.50 g, 66%) from 3-bromo-2-(9*H*-carbazol-9-yl)benzonitrile (**1**) (16.38 g, 47.18 mmol) and dibenzo[*b*,*d*]furan-2-ylboronic acid (10.00 g, 47.18 mmol) using a procedure analogous to that used for 2,3′-di(9*H*-carbazol-9-yl)-[1,1′-biphenyl]-3-carbonitrile (**H1**). **^{1}H NMR** (500 MHz, CD_2Cl_2): δ 8.04 (d, J = 7.8 Hz, 2H), 8.01 (dd, J = 7.9, 1.5 Hz, 2H), 7.96 (dd, J = 7.8, 1.5 Hz, 2H), 7.79 (t, J = 7.8 Hz, 1H), 7.59 (d, J = 1.9 Hz, 1H), 7.55 (d, J = 7.7 Hz, 1H), 7.45 (d, J = 8.2 Hz, 1H), 7.40 (td, J = 7.2, 1.2 Hz, 1H), 7.35 (td, J = 7.2, 1.1 Hz, 2H), 7.21 – 7.28 (m, 3H overlap), 7.15 (d, J = 8.6 Hz, 1H), 7.04 – 7.09 (m, 3H overlap); **^{13}C NMR** (126 MHz, CD_2Cl_2): δ 156.9, 156.3, 144.1, 141.4, 137.9,136.9, 133.9, 130.3, 128.0, 127.3, 126.7, 124.7, 124.1, 123.9, 123.4, 121.0, 120.9, 120.6, 116.6, 115.7, 112.1, 111.8, 110.2; **LCMS-IT-TOF**: 435.16 [(M+H)+]. See Figure S15 and S16.

Synthesis of H4: 8-(2-(9*H*-Carbazol-9-yl)-3-cyanophenyl)dibenzo[*b*,*d*]furan-2-carbonitrile (**H4**) was obtained as white crystal (4.50 g, 45%) from 2-(9*H*-carbazol-9-yl)-3-(4,4,5,5-tetramethyl-1,3,2-dioxaborolan-2-yl)benzonitrile(**2**) (12.87 g, 32.64 mmol) and 8-bromodibenzo[*b*,*d*]furan-2-carbonitrile (5.92 g, 21.76 mmol) using a procedure analogous to that used for 2,3′-di(9*H*-carbazol-9-yl)-[1,1′-biphenyl]-3-carbonitrile (**H1**).**^{1}H NMR** (500 MHz, CD_2Cl_2): *δ* 8.03 (d, *J* = 7.8 Hz, 2H), 7.99 - 8.01 (m, 2H overlap), 7.86 (d, *J* = 1.4 Hz, 1H), 7.82 (t, *J* = 7.8 Hz, 1H), 7.68 (dd, *J* = 8.5, 1.6 Hz, 1H), 7.62 (d, *J* = 1.6 Hz, 1H), 7.53 (d, *J* = 8.5 Hz, 1H), 7.35 (td, *J* = 7.3, 1.2 Hz, 2H), 7.23 (t, *J* = 7.5 Hz, 2H), 7.20 (d, *J* = 8.6 Hz, 1H), 7.13 (dd, *J* = 8.6, 6.8 Hz, 1H), 7.07 (d, *J* = 8.0 HZ, 2H);**^{13}C NMR** (126 MHz, CD_2Cl_2): *δ* 158.7, 156.8, 143.5, 141.4, 138.01, 136.8, 134.2, 133.2, 131.8, 130.5, 128.9, 126.8, 125.7, 125.1, 123.9, 123.2, 121.1, 121.0, 119.4, 116.5, 115.8, 113.4, 112.3, 110.1, 107.4; **LCMS-IT-TOF**: 460.14 [$(M+H)^+$]. See Figure S17 and S18.

Synthesis of Precursor 1: A solution of 9*H*-carbazole (36.12 g, 216.00 mmol) in anhydrous N,N-dimethylformamide (DMF) was added dropwise into dispersion of sodium hydride (60%, 8.64 g, 216.00 mmol) in anhydrous DMF in an ice bath. After stirring for 1 h, 3-bromo-2-fluorobenzonitrile (47.52 g, 237.60 mmol) was dissolved in anhydrous DMF and added to the stirred reaction mixture under a nitrogen atmosphere. The reaction mixture was stirred at 130 °C for 15 h. After allowing it to cool to room temperature, the reaction mixture was extracted with dichloromethane and water. The organic layer was dried using magnesium sulfate and evaporated with a rotary evaporator. The crude product was purified by column chromatography using dichloromethane/*n*-hexane (1:2) and then dried under vacuum to give 3-bromo-2-(9*H*-carbazol-9-yl)benzonitrile (**1**) (51.50 g, 69%).

Synthesis of Precursor 2: A mixture of 3-bromo-2-(9H-carbazol-9-yl)benzonitrile (1) (34.35 g, 98.91mmol), bis(pinacolato)diboron (37.68 g, 148.37mmol), potassium acetate (29.12 g, 296.74mmol), and [1,1′-bis(diphenylphosphino)ferrocene]dichloropalladium(II) (7.24 g, 9.89mmol) in 250 mL of anhydrous DMF was stirred at 150 °C for 12h. After allowing it to cool to room temperature, the reaction mixture was extracted with dichloromethane and water. The organic layer was dried using magnesium sulfate and evaporated with a rotary evaporator. The crude product was purified by column chromatography using dichloromethane/n-hexane (1:1) and then dried under vacuum to give 2-(9H-carbazol-9-yl)-3-(4,4,5,5-tetramethyl-1,3,2-dioxaborolan-2-yl)benzonitrile(2) (25.10 g, 64%).

Synthesis of Precursor 3: A mixture of 2-(9*H*-carbazol-9-yl)-3-(4,4,5,5-tetramethyl-1,3,2-dioxaborolan-2-yl)benzonitrile (**2**) (10.02 g, 25.42mmol), 1-bromo-3-iodobenzene (10.79 g, 38.14mmol), tripotassium phosphate (16.19 g, 76.27 mmol), SPhos (1.04 g, 2.54 mmol), and bis(dibenzylideneacetone)palladium(**0**) (0.73 g, 1.27 mmol) in 64 mL of toluene/water (10:1) was stirred at 100 °C for 13 h. The reaction mixture was extracted with toluene and water. The organic layer was dried using magnesium sulfate and evaporated with a rotary evaporator.

The crude product was purified by column chromatography using dichloromethane/n-hexane (1:1) and then dried under vacuum to give 3′-bromo-2-(9*H*-carbazol-9-yl)-[1,1′-biphenyl]-3-carbonitrile (**3**) (5.00 g, 45%).

Synthesis of Precursor 4: A mixture of carbazole (12.97 g, 77.59 mmol), 1-bromo-3-fluorobenzene (20.37 g, 116.38 mmol), and cesium carbonate (50.56 g, 155.18 mmol) in 190 mL of dimethylacetamide was stirred at 160 °C for 12 h. After allowing it to cool to room temperature, the reaction mixture was extracted with dichloromethane and water. The organic layer was dried using magnesium sulfate and evaporated with a rotary evaporator. The crude product was purified by column chromatography using dichloromethane/*n*-hexane (1:5) and then dried under vacuum to give 9-(3-bromophenyl)-9*H*-carbazole(**4**) (22.00 g, 88%).

Synthesis of Precursor 5: 9-(3-(4,4,5,5-Tetramethyl-1,3,2-dioxaborolan-2-yl)phenyl)-9*H*-carbazole (**5**) was obtained as white crystal (16.10 g, 81%) from 9-(3-bromophenyl)-9*H*-carbazole (**4**) (17.45 g, 54.16 mmol) using a procedure analogous to that used for 2-(9*H*-carbazol-9-yl)-3-(4,4,5,5-tetramethyl-1,3,2-dioxaborolan-2-yl)benzonitrile (**2**).

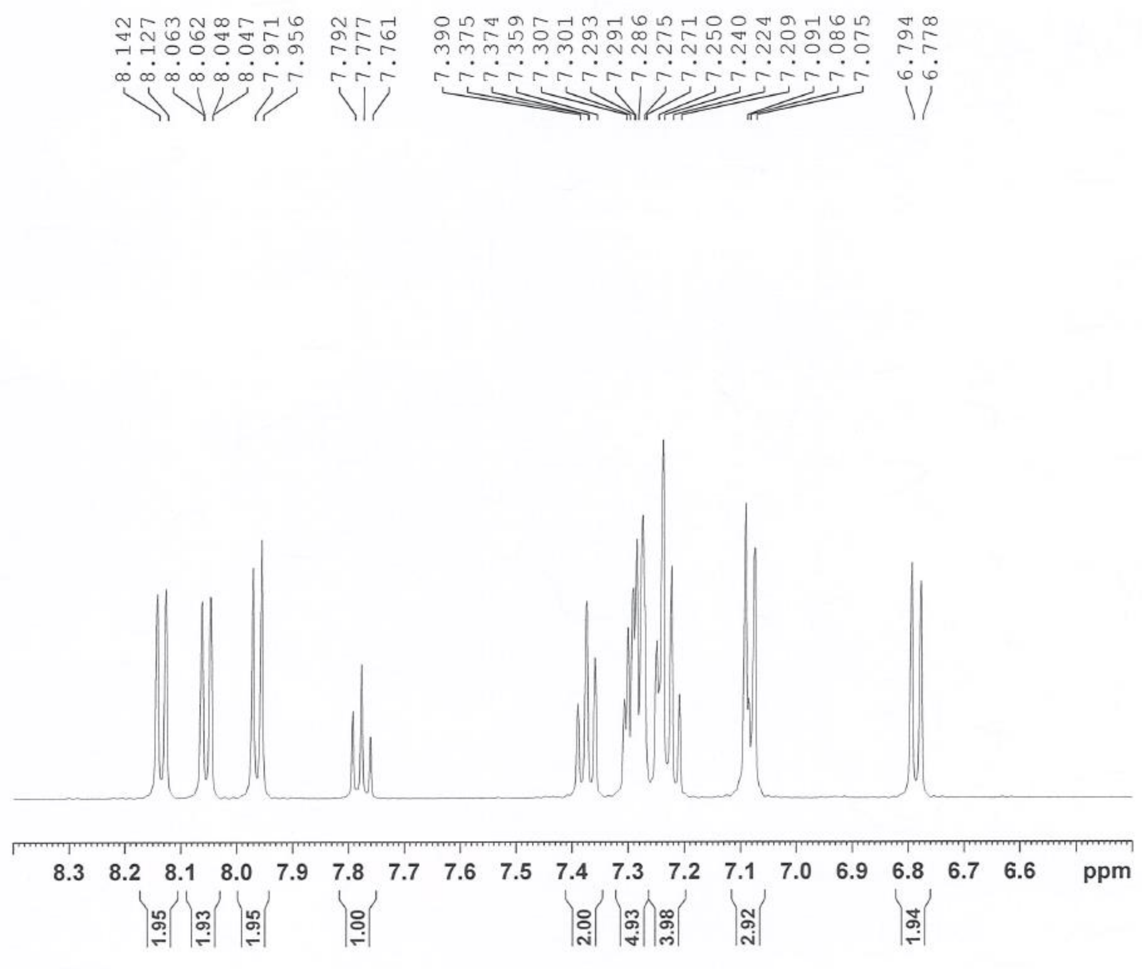

Figure S11. ^{1}H NMR spectra of H1.

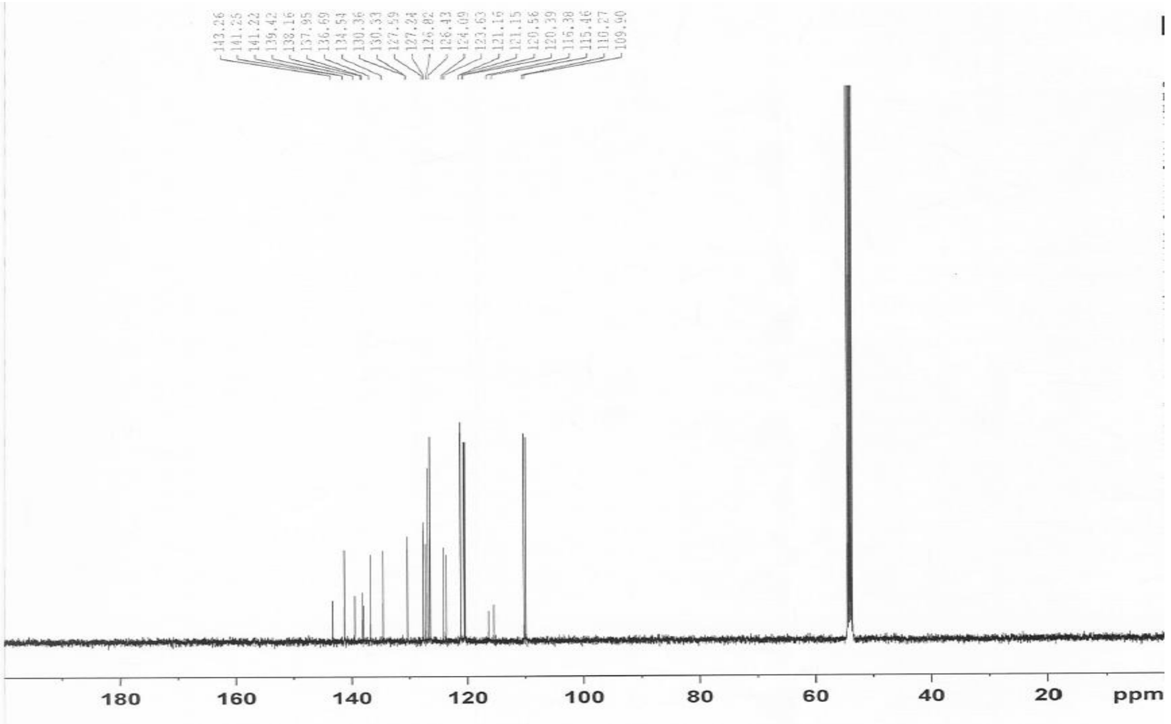

Figure S12. ^{13}C NMR spectra of H1.

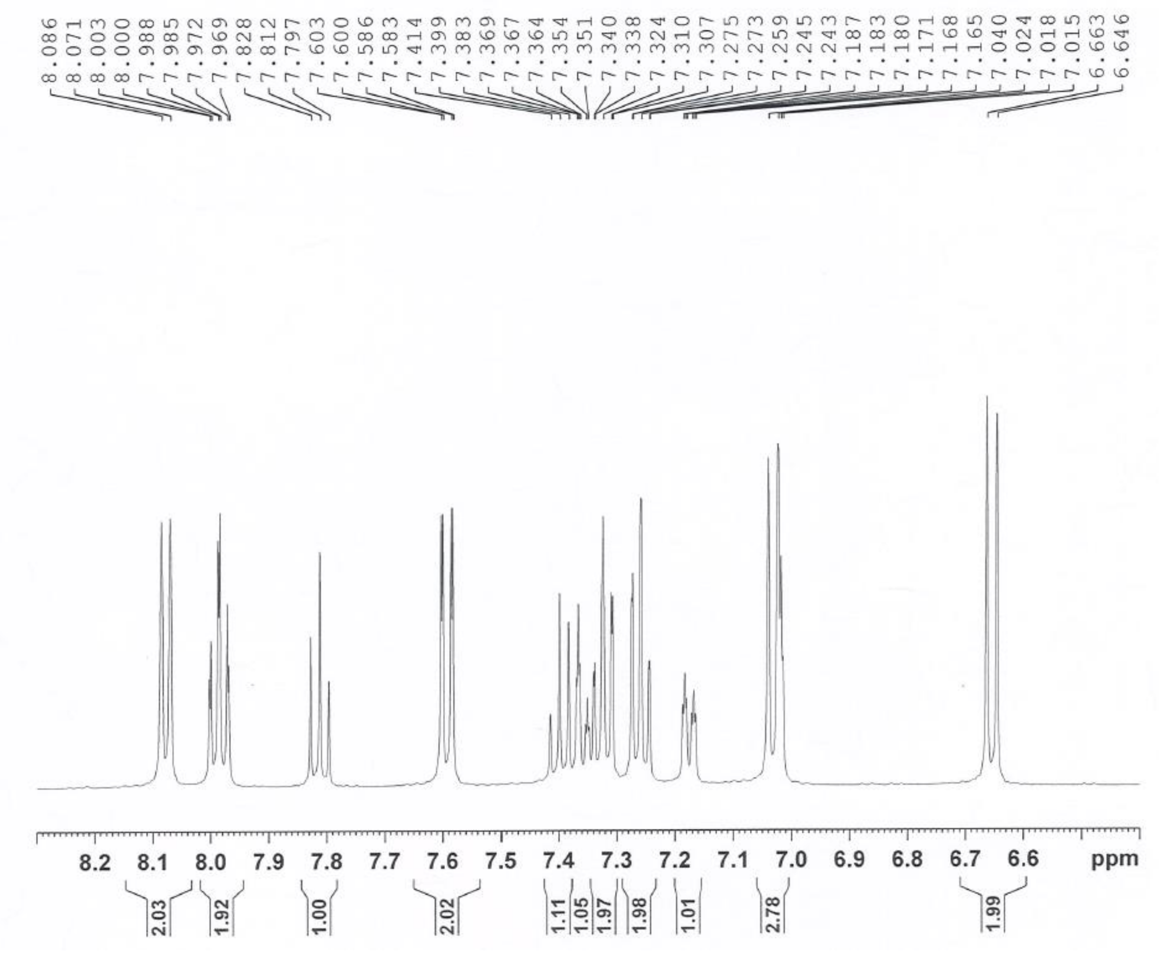

Figure S13. ^{1}H NMR spectra of H2.

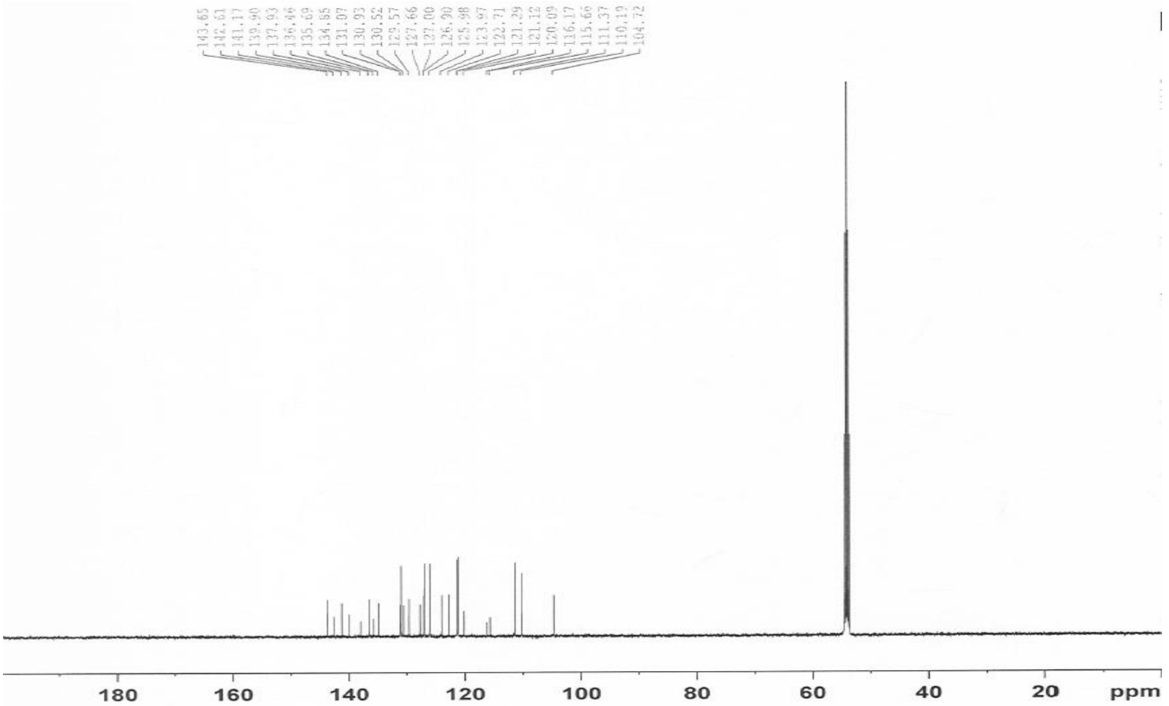

Figure S14. ^{13}C NMR spectra of H2.

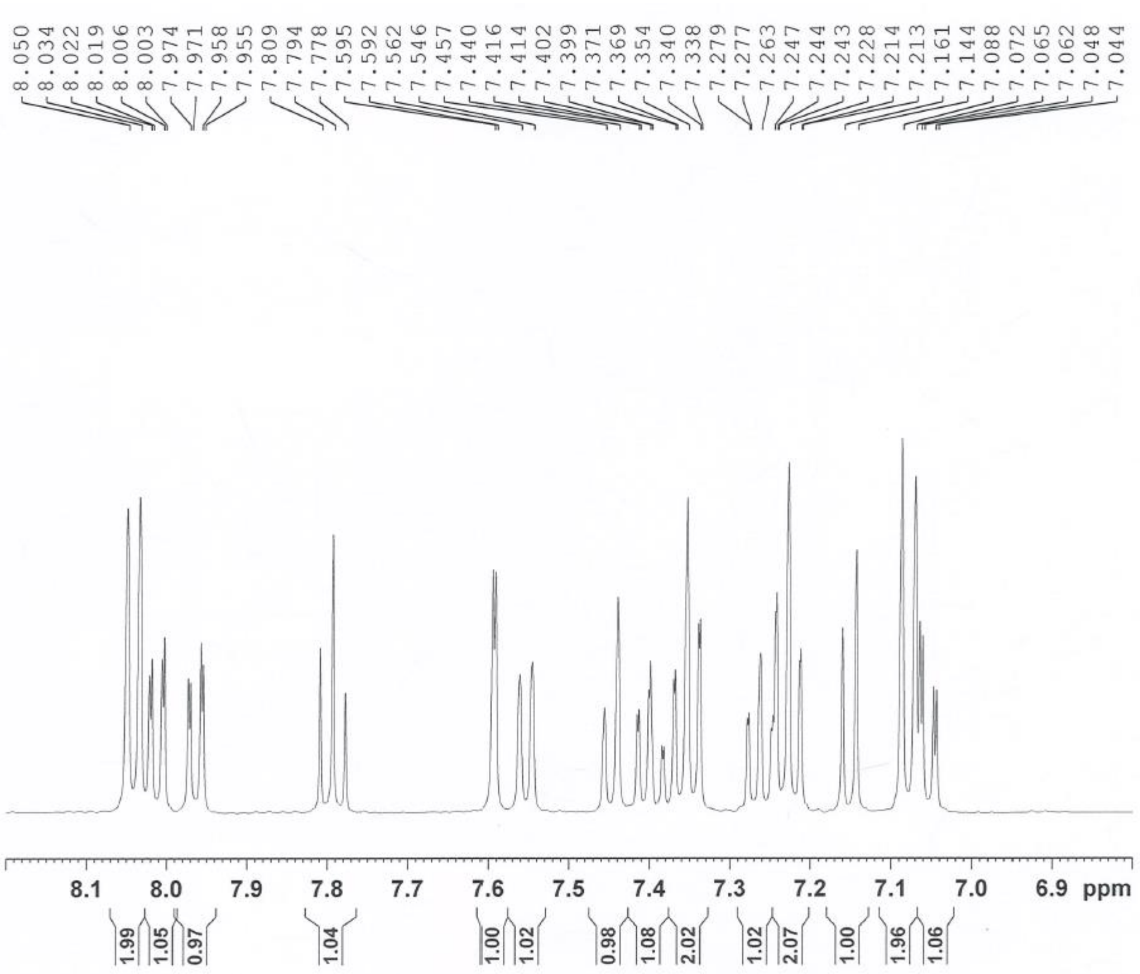

Figure S15. ^{1}H NMR spectra of H3.

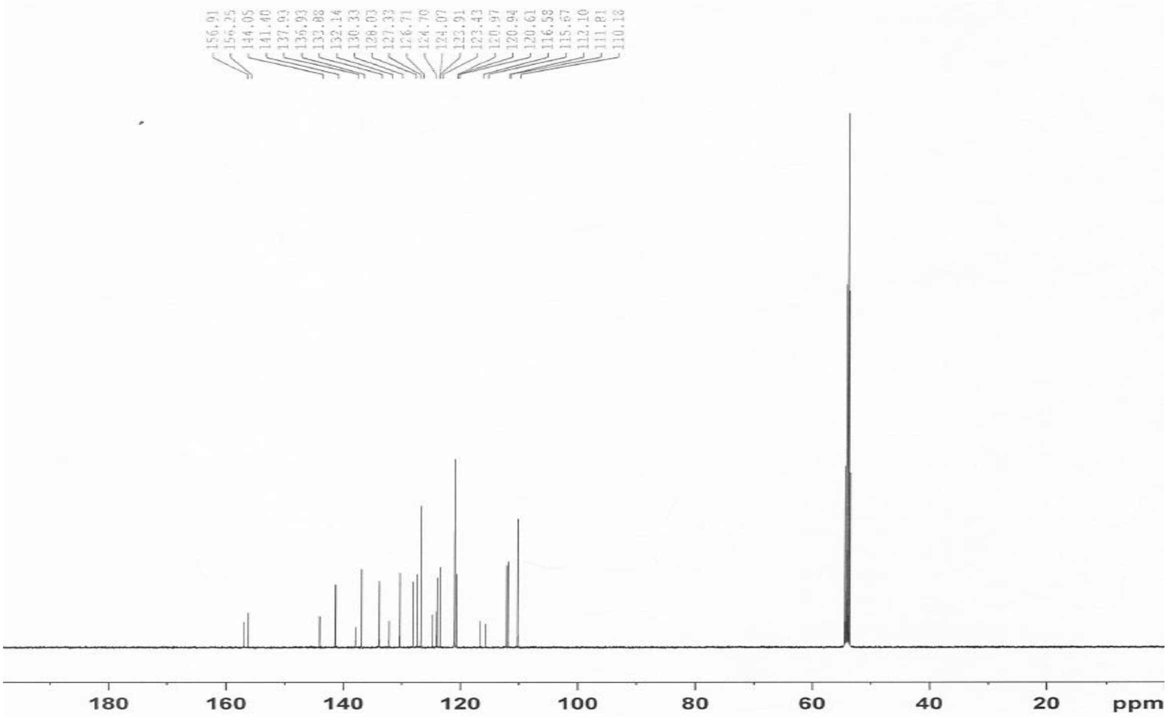

Figure S16. ^{13}C NMR spectra of H3.

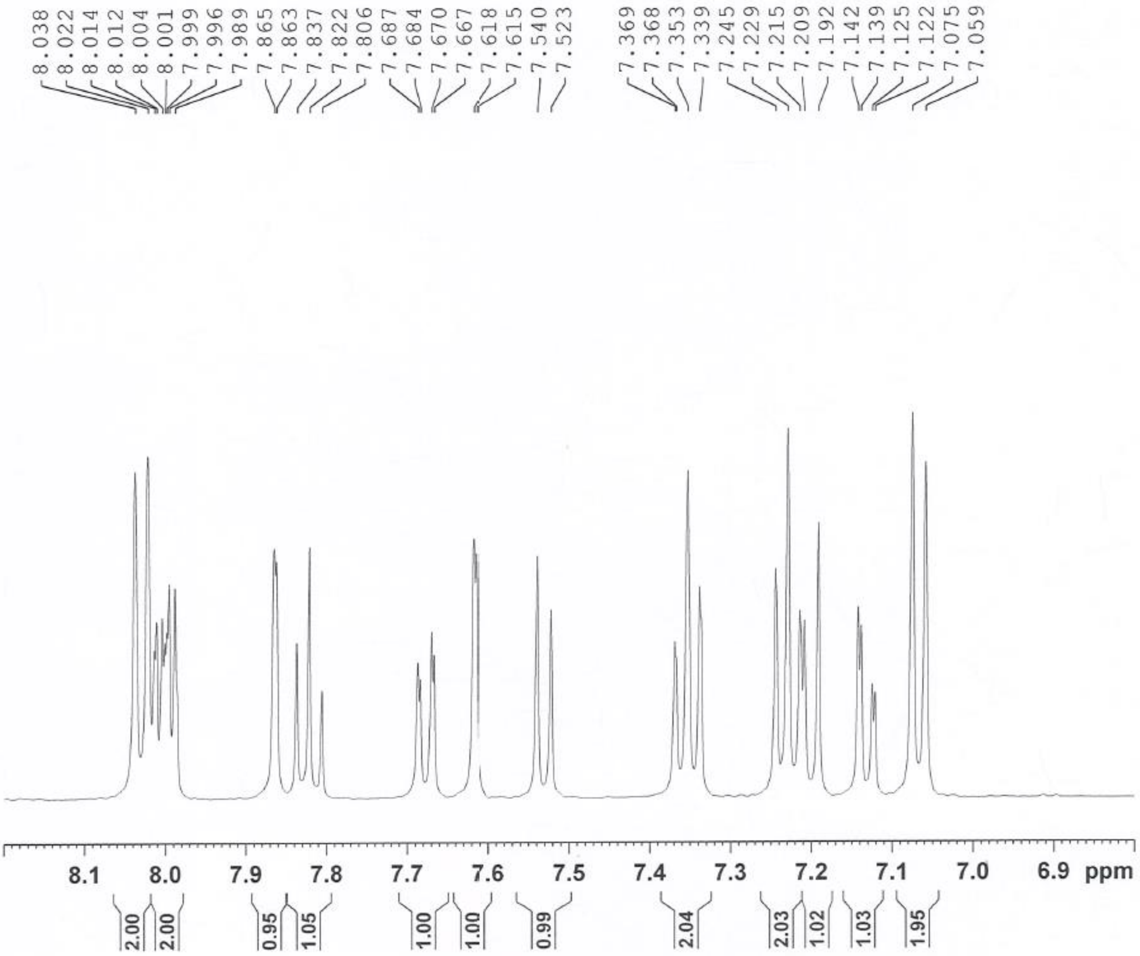

Figure S17. ^{1}H NMR spectra of H4.

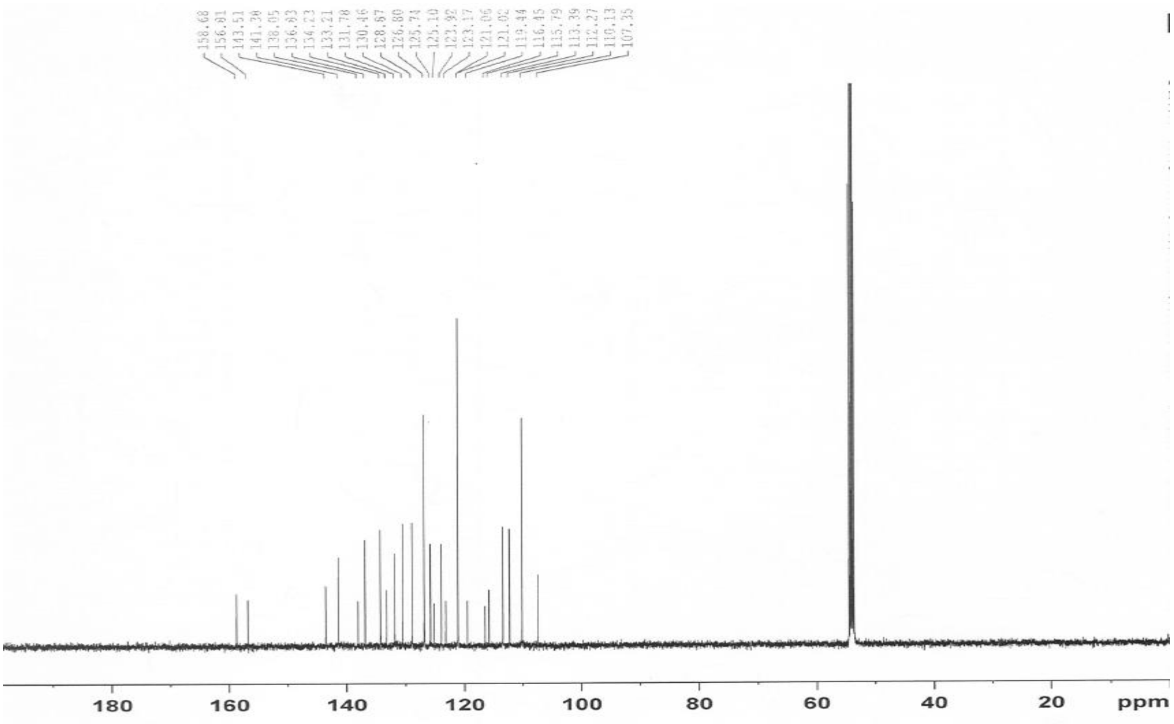

Figure S18. ^{13}C NMR spectra of H4.

Table S1. Physical properties of H1, H2, H3 and H4.

Host	S_1 [eV]	T_1 [eV]	T_g [a)] [℃]	T_d [b)] [℃]
H1	3.39	3.06	99	300
H2	3.46	3.08	147	362
H3	3.42	3.11	83	242
H4	3.39	3.06	112	303

[a)] S_1: Energy of the lowest singlet excited state calculated from the onset of the fluorescence spectrum.

[b)] T_1: Energy of the lowest triplet excited state calculated from the onset of the phosphorescence spectrum.

[c)] T_g: Temperature for glass-transition.

[d)] T_d: Temperature for 0.1 wt% loss.

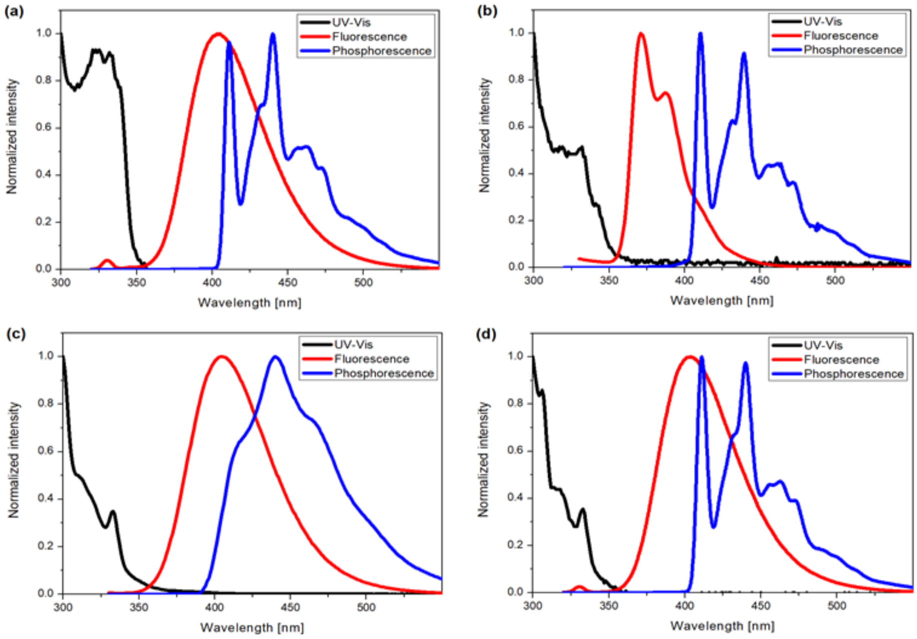

Figure S19. UV-Vis and PL spectra of (a) H1, (b) H2, (c) H3 and (d) H4 in tetrahydrofuran. PL spectra were used for calculation of S_1 and T_1 which are shown in Table S1.

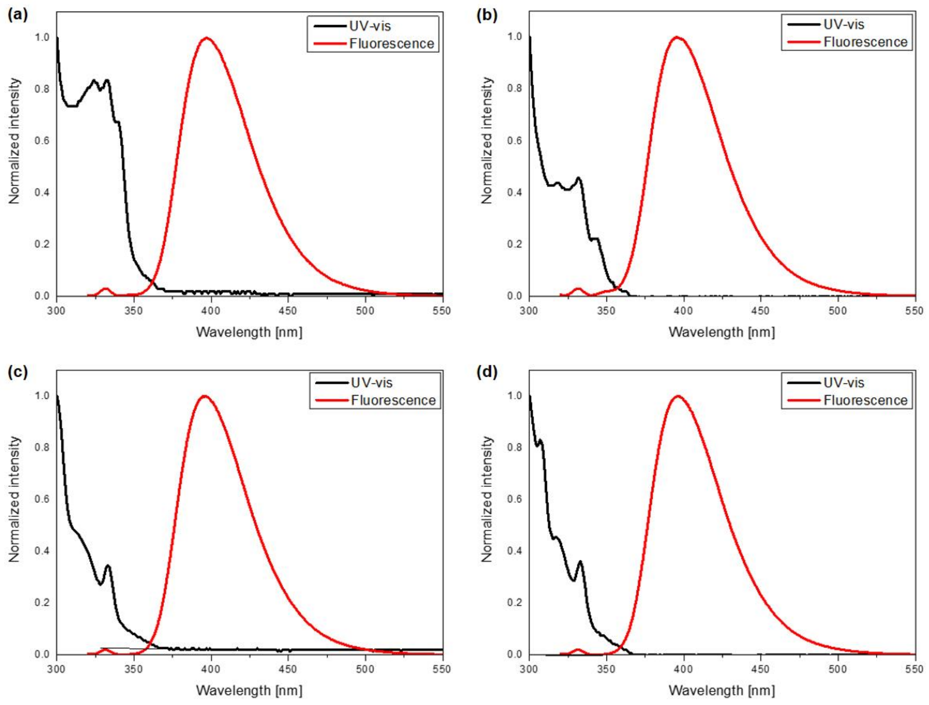

Figure S20. UV-Vis and PL spectra of (a) H1, (b) H2, (c) H3 and (d) H4 in toluene.

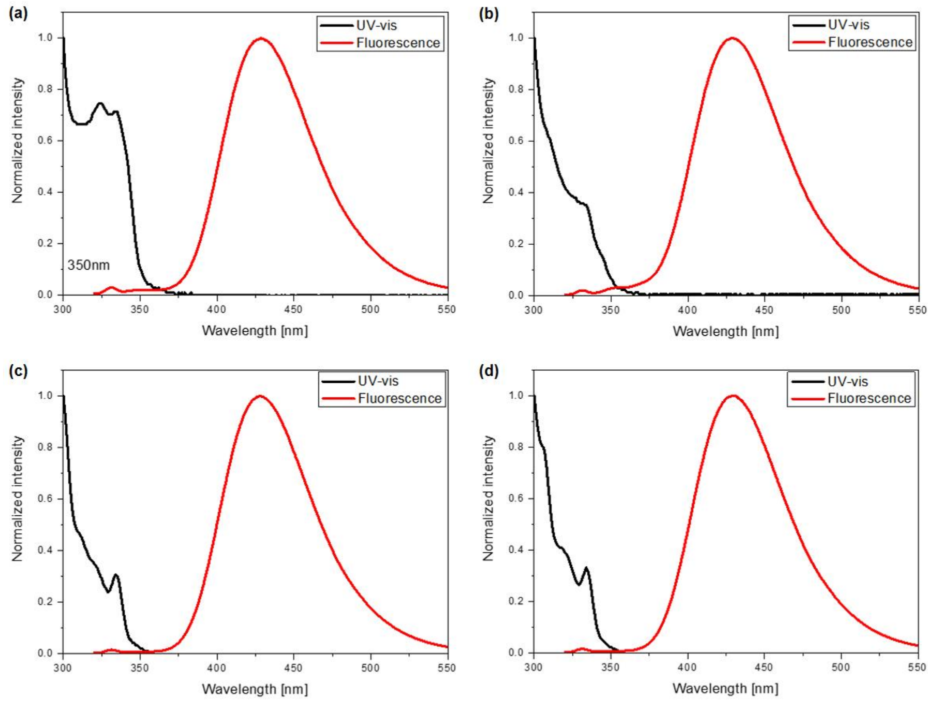

Figure S21. UV-Vis and PL spectra of (a) H1, (b) H2, (c) H3 and (d) H4 in dimethyl sulfoxide.
